# Concurrent Assessment of Synthetic and Natural Compounds on the Proliferation of *Toxoplasma gondii* in In Vitro Models

**DOI:** 10.3390/tropicalmed10120349

**Published:** 2025-12-13

**Authors:** Alejandro Zamora-Vélez, Derly Lorena Vanegas, María Camila Fernández, Gerardo Ramos, Edwar Cortés, Ailan Farid Arenas, Néstor Cardona, Jessica Palacio-Rodriguez, Juan David Valencia-Hernandez, Luz Angela Veloza, Juan Carlos Sepúlveda-Arias, Jorge Enrique Gómez-Marín

**Affiliations:** 1 GEPAMOL (Grupo de Estudio en Parasitologia Molecular), Center for Biomedical Research (CIBM), Faculty of Health Sciences, University of Quindío, Armenia 630004, Colombia; oazamora@uniquindio.edu.co (A.Z.-V.); dlvanegas@uniquindio.edu.co (D.L.V.); mariac.fernandezq@uqvirtual.edu.co (M.C.F.); afarenas@uniquindio.edu.co (A.F.A.); nicardona@uniquindio.edu.co (N.C.); japalacior@uqvirtual.edu.co (J.P.-R.); juand.valenciah@uniquindio.edu.co (J.D.V.-H.); 2 GICOC (Grupo de Investigación en Compuestos Organometálicos y Catálisis), Programa de Química, Facultad de Ciencias Básicas, Universidad del Quindio, Armenia 630004, Colombia; garamos@uniquindio.edu.co; 3 GIPRONUT (Grupo de Investigación en Productos Naturales), Programa de Química, Facultad de Ciencias, Universidad del Tolima, Ibagué 730002, Colombia; ecortesgo@ut.edu.co; 4 Faculty of Dentistry, Universidad Antonio Nariño, Armenia 630004, Colombia; 5 Grupo Polifenoles, Facultad de Tecnología, Escuela de Química, Universidad Tecnológica de Pereira, Pereira 660003, Colombia; lveloza@utp.edu.co; 6 Grupo Infección e Inmunidad, Facultad de Ciencias de la Salud, Universidad Tecnológica de Pereira, Pereira 660003, Colombia; jcsepulv@utp.edu.co

**Keywords:** *Toxoplasma*, *Tabebuia*, thiazolidines, pyrazolidines

## Abstract

Concurrent evaluation of the antiparasitic efficacy of synthetic and natural compounds can provide novel insights into the development of anti-*Toxoplasma* drugs. We assessed 16 synthetic compounds and two fractions derived from the leaves of *Tabebuia rosea* and *Tabebuia chrysantha* tree species for their in vitro activity against live parasites, employing strains that express green fluorescent protein and specific identification of bradyzoites using an anti-BAG1 monoclonal antibody. This study successfully identified several promising synthetic compounds with potent anti-*Toxoplasma* activity and favorable in vitro selectivity profiles, notably pyrazoline 2 and thiazolidinone 9. One thiazolidinone compound exhibited significant activity against extracellular tachyzoites, whereas one tree fraction demonstrated excellent activity against both tachyzoites and bradyzoites. Additionally, their in silico ADMET properties suggest their potential for good in vivo performance and CNS penetration. Although the natural extracts showed less potency in their crude form, they provide a basis for future purification efforts. The simultaneous evaluation of compounds sourced from diverse discovery pipelines can offer valuable insights into the development of drugs that target various biological pathways.

## 1. Introduction

*Toxoplasma gondii* is the etiological agent responsible for toxoplasmosis, a zoonotic disease of considerable medical and veterinary significance. Wild and domestic felids are the sole definitive hosts capable of shedding oocysts in their feces [1]. Infection commonly occurs through several pathways, including the ingestion of oocysts, which are in the environmentally resistant stage found in contaminated water, soil, and food; the consumption of undercooked or raw meat containing tissue cysts with bradyzoites; and congenital transmission [1]. Seroprevalence studies conducted worldwide indicate that 15–85% of the human population is infected with *T. gondii* [2]. Nonetheless, the epidemiological management and the development of novel chemotherapeutic agents characterized by low toxicity and high specificity remain significant challenges [3]. The treatment for toxoplasmosis involves a regimen of pyrimethamine and sulfadiazine, which inhibit nucleic acid synthesis and exert a parasitostatic effect, limiting the replication of *T. gondii* tachyzoites [3]. However, this treatment induces severe adverse effects, including hypersensitivity, hematological toxicity, and bone marrow suppression, which may necessitate dose reduction or therapy discontinuation [3,4]. A significant issue with current pharmacological compounds is their ability to control tachyzoite proliferation while failing to eradicate tissue cysts containing bradyzoites. These bradyzoites persist throughout the individual’s lifetime and are responsible for clinical recurrences in cases of ocular toxoplasmosis or systemic infections due to reactivation in immunosuppressed individuals [5].

Researchers in countries where tropical diseases are endemic face substantial challenges in their search for novel treatments. The scarcity of resources and reliance on postgraduate students to initiate the evaluation of new compounds result in inconsistencies in outcomes and a lack of continuity in the development and acquisition of new pharmacological entities. This situation has resulted in a significant loss of scientific investment in these nations, posing a significant obstacle to achieving health autonomy. Numerous compounds have been evaluated in toxoplasmosis research through individual studies and high-throughput screening processes over several decades. The findings of these investigations have been disseminated across an extensive corpus of scientific literature and archived in various databases [6]. The exact cumulative number has not been centrally tracked or publicly compiled into a single comprehensive figure. Research papers typically focus on specific libraries of compounds, which can range in size from a few dozen to thousands. One systematic review mentioned evaluating 80 clinically available drugs and several new compounds [7].

Our research group has collaborated with groups in Colombia and the United States to identify new compounds for chemical synthesis and to examine natural compounds with anti-*Toxoplasma* activity [8,9]. One significant challenge is the use of diverse methodologies to assess anti-*Toxoplasma* activity, which may hinder the comparison and identification of the critical IC50 value needed to guide the selection of optimal compounds for further characterization, which is potentially costly.

Among compounds of interest, those containing a pyrazole ring have demonstrated a range of biological activities, including antimicrobial, antifungal, leishmanicidal, antiviral, anti-Chagas, pesticidal, antihyperglycemic, anti-inflammatory, and antitumor effects [10,11]. Derivatives of pyrazolopyrimidine and 5-aminopyrazole-4-carboxamide have been employed to inhibit the calcium-dependent protein kinase 1 of *T. gondii* (TgCDPK1), demonstrating low nanomolar activity against the enzyme, with an EC50 ranging from 89 nM to 2.250 nM [12]. Although derivatives of the pyrazoline ring, which is structurally like the pyrazole ring with five members and two nitrogen atoms but containing one double bond, have not been evaluated for their efficacy against *T. gondii* proliferation, they have demonstrated biological activities such as antimalarial, antibacterial, and antifungal effects [13,14,15].

Other compounds with evaluated biological activity against *T. gondii* have been associated with thiazolidinone nuclei. Modifications at the 2 and 5 positions of the 4-thiazolidinone ring are crucial for enhancing the biological activity of compounds containing this structure. Furthermore, the presence of a hydrazone group at these positions on the heterocyclic ring has been shown to enhance the efficacy of 4-thiazolidinones against *T. gondii*. Compounds featuring this group have demonstrated significant effectiveness in inhibiting host cell invasion and parasite replication while maintaining low cytotoxicity at micromolar concentrations [16]. Hybrid molecules combining thiazolidinone with pyridine-4-carbohydrazone (PCH) or thiosemicarbazone (TSC) structures obtained an IC50 between 8.3 and 39 µg/mL and SI between 9.92 and 148.28 for anti-*T. gondii* activity [17]. One new 4-thiazolidinone tested on invasion and growth of *T. gondii* reached a selective index (SI) of 1000 [8].

In addition to these synthetic compounds, we also evaluated plant extracts. Our previous study indicated that the chloroform-derived (CHCl3) fraction of the leaves of *Tabebuia rosea* and *T. chrisantha* exhibits anti-*Toxoplasma* activity in vitro against tachyzoites [9]. The fraction exhibited a mean cytotoxic concentration (CC50) of 50 μg/mL on HFF cells, a mean inhibitory concentration (IC50) of 6.8 μg/mL, and a therapeutic index (TI) of 7.3, with β-amyrin identified as the major component [9].

In light of recent advancements in the development of novel compounds for potential toxoplasmosis treatment, this study evaluated the anti-*T. gondii* activity of newly synthesized compounds with pyrazoline, pyrazole, and thiazolidinone nuclei, as well as fractions derived from *Tabebuia*, using consistent methodologies and study models.

## 2. Materials and Methods

### 2.1. Synthesis of Pyrazole, Pyrazoline and Thiazolidinone Derivatives Compounds

The synthesis experiments are described in detail in the Supplementary Material. In summary, four pyrazole, four pyrazoline, and eight thiazolidinone derivatives were synthesized. For the pyrazole precursors, cyanoacetyl indole and the aldehyde of interest were reacted at a 1:1 ratio. The mixture was covered with ethanol and two drops of piperidine were added, followed by stirring at room temperature for 12 h or until the reaction reached completion. The progress of the reaction was monitored by thin-layer chromatography (TLC, New York, NY, USA). To facilitate product formation, four drops of hydrazine were added, and the mixture was stirred for 3–4 h at 80 °C. All reactions were monitored by thin-layer chromatography (TLC) on silica gel 60 F254 (Merck, Rahway, NJ, USA). The chemicals, solvents for synthesis, and spectral-grade solvents were procured from commercial suppliers (Sigma Aldrich, Burlington, MA, USA) and Alfa Aesar (Thermo Fisher Scientific, Waltham, MA, USA) and were used without further purification. The melting points were determined using a fusiometer (IA9100 Electrothermal, Fisher Scientific, Waltham, MA, USA).

### 2.2. Fraction from Tabebuia rosae and T. chrysantha Tree

Previous research evaluated the activity against tachyzoites from 12 fractions of *Tabebuia chrysantha* and 14 fractions of *T. rosea* [9]. In this study, we extended previous observations and evaluated those with increased activity from both species of *Tabebuia*. All experimental protocols were approved by the Bioethics Committee of the Faculty of Health Sciences at Universidad del Quindío (Act No. May 18 29, 2019). The leaves of *T. rosea* (Bertol.) DC were collected on the campus of the Universidad Tecnológica de Pereira, located at a latitude of 4°79′23.39″ N and a longitude of 75°69′0742″. The plants were identified at the National Herbarium of Colombia (Voucher No. COL 582,577 for *T. rosea*). The collection and processing of the material were conducted under the framework collection permit number 237/2018 (Otrosí No. 4) issued by the “Ministerio de Ambiente y Desarrollo Sostenible.” The chloroform fractions from the leaves of *T. rosea* (Tr-H-CHCl3) and *T. chrysantha* (Tc-HCHCL3) were obtained as previously described [17]. The fractions were characterized using thin-layer chromatography (TLC) in both the normal phase (silica) and reverse phase (RP-18) with hexane–ethyl acetate (7:3) and water–isopropanol (7:3) elution systems. The chromatographic plates were developed using aluminum chloride (AlCl_3_, Sigma Chemical Co., Saint Louis, MO, USA), ferric chloride (FeCl_3_, Sigma Chemical Co., Saint Louis, MO, USA), and a mixture of vanillin–phosphoric acid (H_3_PO_4_) for the detection of flavonoids, phenols, and lignans. Potassium hydroxide (KOH, Merck, Darmstadt, Germany) in analytical-grade ethanol was used to detect anthrones, quinones, and coumarins; a mixture of anisaldehyde–acetic acid–sulfuric acid (H_2_SO_4_) with vanillin–phosphoric acid (H_3_PO_4_) was used to detect iridoids; a mixture of anisaldehyde–acetic acid–sulfuric acid (H_2_SO_4_) with antimony chloride (SbCl_3_) was used to detect saponins and triterpenes; oleum (Sigma Chemical Co., Saint Louis, MO, USA) was used to detect sesquiterpene lactones; 2,4-dinitrophenylhydrazine was used to determine aldehydes and ketones; and the Liebermann–Burchard reagent was used to detect terpenes and steroids. The extracts and fractions exhibited specific colors when reacted with the developing reagents for each test. The absence or presence of this color was considered a negative (−) or positive (+) result for the presence of these phytochemical components. The results of the phytochemical analyses are presented in Supplementary Material.

Preliminary phytochemical analysis of the extracts prepared from the inner bark and leaves of *T. rosea* revealed the presence of flavonoids, lignans, terpenes, aldehydes, ketones, and unsaturated fatty acids. In contrast, preliminary phytochemical analysis of the extracts prepared from the inner bark and leaves of *T. chrysantha* revealed the presence of lignans, coumarins, terpenes, sterols, iridoids, triterpenes, saponins, and unsaturated fatty acids in all the extracts.

### 2.3. Host Cell Lines and Cultivation of T. gondii Parasites

All cultures were maintained in a humidified incubator at 37 °C with 5% CO_2_. Specifically, Vero cells, derived from the kidney tissue of a normal adult African green monkey (ATCC No: CCL-81), and HFF (Human Foreskin Fibroblast-ATCC No: SCRC-1041) monolayers were cultured as monolayers in Dulbecco’s Modified Eagle’s Medium (DMEM). This medium was supplemented with 25 mM glucose, 4 mM L-glutamine, 1 mM sodium pyruvate, 100 U/mL penicillin, 100 μg/mL streptomycin, and 10% heat-inactivated bovine serum. The *T. gondii* RH GFP strain, tagged with green fluorescent protein and generously provided by Dr. David Sibley from the University of Washington, United States, was consistently maintained in vitro in Vero monolayers. These monolayers were grown in DMEM supplemented with 25 mM glucose, 4 mM L-glutamine, 1 mM sodium pyruvate, 100 U/mL penicillin, 100 μg/mL streptomycin, and 1% heat-inactivated fetal bovine serum (tachyzoite medium). Freshly egressed parasites were transferred to new Vero monolayers for continued culturing.

### 2.4. Cytotoxicity Evaluation of the Selected Compounds In Vero Cells by Alamar Blue Assay

The Vero cell line was seeded in 96-well plates at approximately 20,000 cells per well until approximately 100% confluence was reached. These cells were treated in triplicate with the compounds at seven concentrations and 1:2 dilutions, starting at 160 μM and decreasing to a minimum concentration of 1.25 μM for 24 h. All compounds were evaluated at elevated concentrations, diluted in 1% DMSO, to determine the maximum concentration at which they could remain in solution for experimental purposes. It was observed that most compounds were not fully soluble at 320 µM. The subsequent lower concentration at which solubility was consistently achieved was 160 µM. Consequently, all further experiments were initiated from this concentration. The controls for this experiment were untreated cells (UC), 10 μM pyrimethamine (Pyr), 1% DMSO, and 10% Triton X. After 24 h, resazurin was added at a concentration of 0.44 mM and incubated at 37 °C with 5% CO_2_. Four hours later, fluorescence was measured using a BioTek Synergy HTX plate reader at 530/25 nm excitation and 590/25 nm emission. Host cell viability was determined by comparing the treatments without the compound, with 100% viability. A color change in the growth control well to pink indicated proper cell growth, and no color change in the sterile control well indicated the absence of contaminants.

### 2.5. T. gondii RH GFP Tachyzoite Viability Assay

Approximately 10,000 tachyzoites of the *T. gondii* RH GFP strain were quantified using a hemocytometer and subsequently inoculated into Vero cells at full confluence per well in a 96-well plate using a medium conducive to parasite maintenance. Following our laboratory protocols and the methodologies outlined in previous studies [10], we routinely utilize an inoculum of 10,000 parasites for the *Toxoplasma gondii* RH-GFP tachyzoite viability assay, which is conducted over a 72 h duration. This parasite density ensures reliable detection of tachyzoite growth and viability while minimizing the likelihood of detachment or any degree of disruption to the host cell monolayer. Concurrently, derivatives of pyrazoline, pyrazole, and thiazolidinone nucleus compounds were introduced at seven concentrations, with 1:2 dilutions commencing at 160 μM and decreasing to a minimum concentration of 1.25 μM in triplicate. The controls employed in this experiment included non-infected cells (NC), 10 μM pyrimethamine (Pyr), 1% dimethyl sulfoxide (DMSO), and infected cells without treatment (IC). Seventy-two hours post-infection, each well in the 96-well plate was examined using a 10× objective on the EVOS FL Color Imaging System with the GFP fluorescent light cube, and images were captured approximately at the center of each well. The parasites that glowed green were considered viable, and the percentage of tachyzoite viability was compared with that of the untreated parasites. Subsequently, images were recorded, contrast was adjusted, and a semiquantitative digital analysis was conducted using ImageJ v1.54d and the triangle auto-threshold method to determine the number of viable GFP fluorescent tachyzoites per image, as previously described [8]. The percentage of viable parasites was calculated using the formula: Fluorescence of the sample with treatment URF × 100/fluorescence of the control without treatment, where URF = unit relatives of fluorescence calculated with the formula: (sample fluorescence/fluorescence in blank well) × 100.

### 2.6. Assessment of T. gondii RH GFP Tachyzoite Viability Following a 24 h Pretreatment of Host Cells

Vero cells, at full confluence in each well of a 96-well plate, were initially treated for 24 h with compounds identified as “hits,” which exhibited a half-maximal inhibitory concentration (IC50) of less than 10 µM across seven concentrations with 1:2 dilutions, starting at 160 μM and decreasing to a minimum concentration of 1.25 μM. Subsequently, 10,000 *T. gondii* RH GFP tachyzoites were introduced into each well. The experimental controls included non-infected cells (NC), 10 μM pyrimethamine (Pyr), 1% dimethyl sulfoxide (DMSO), and infected cells without treatment (IC). After 24 h of host cell treatment, the medium was replaced with a compound-free medium for parasite maintenance. Seventy-two hours post-infection, each well of the 96-well plate was examined using a 10× objective on an Evos FL Auto Cell Imaging System microscope (Life Technologies, Carlsbad, CA, USA). A semiquantitative digital analysis was conducted using ImageJ v1.54d and the triangle auto-threshold method to quantify the number of viable GFP fluorescent tachyzoites per image, as previously described [8].

### 2.7. Assessment of the Viability of T. gondii RH GFP Tachyzoite Following a 3 h Pretreatment of Parasites

In this experiment, tachyzoites of *T. gondii* RH GFP strain were subjected to a 3 h treatment with compounds identified as “hits,” characterized by IC50 values below 10 µM across seven concentrations with 1:2 dilutions, starting at 160 μM and decreasing to a minimum concentration of 1.25 μM. Following treatment, tachyzoites were centrifuged at 712× *g* for 10 min, and the supernatant was discarded. Fresh medium devoid of compounds was then added to resuspend the parasites. Subsequently, 10,000 pretreated *T. gondii* RH GFP tachyzoites were introduced into each well of a 96-well plate containing Vero cells at 100% confluence. Seventy-two hours post-infection, each well of the 96-well plate was examined using a 10× objective on an EVOS microscope. Semiquantitative digital analysis was performed using ImageJ v1.54d, employing the triangle auto-threshold method to quantify the number of viable GFP fluorescent tachyzoites per image, as previously described [8].

### 2.8. Half Maximal Cytotoxic Concentration (CC50), Half Maximal Inhibitory Concentration (IC50) and Selectivity Index (SI) Calculation

The normalized viability percentages (Y-values) of viable host cells and viable *T. gondii* GFP RH tachyzoites were calculated from the mean ± standard deviation for each concentration evaluated in triplicate. The concentration values (*X*-axis) were subjected to logarithmic transformation to fit the data to a sigmoidal curve. The half-maximal cytotoxic concentration (CC50) was estimated as the interpolated concentration value on the *X*-axis corresponding to the response midway between the fitted top and bottom plateaus of the curve. Similarly, the half-maximal inhibitory concentration (IC50) was determined as the concentration at which the response was halfway between the top and the bottom plateaus. Data analysis and visualization were conducted using GraphPad Prism version 8.4.3 (GraphPad Software, San Diego, CA, USA). The selectivity index, also known as the Therapeutic Index, was calculated using the formula: SI = CC50/IC50. Notably, the selectivity index was calculated using the maximum tested concentration of CC50.

### 2.9. In Silico Pharmacokinetics, Drug-Likeliness, and Admet Depiction Exploration

To obtain a novel medication with high efficiency and less experimental investigation time, it is mandatory to evaluate their absorption, distribution, metabolism, excretion, and toxicity (ADMET) properties in humans before beginning any clinical trials, verifying the five rules of Lipinski [18]. For this purpose, we predicted in silico the pharmacokinetics and ADMET properties by using pkCSM software [18]

### 2.10. Anti-Bradyzoite Activity of T. rosae Fractions

An in vitro model using antibodies against bradyzoite-specific antigen 1 (BAG1) was used to evaluate the effect of the chloroform-derived fraction of *T. rosea* leaves (Tr-H-CHCl_3_) on the bradyzoite stage of *T. gondii* [19]. HFF cell monolayers were infected with tachyzoites of the Prugniaud (PRU) clonal type II strain of *T. gondii*, kindly donated by the laboratory of Dr. Laura Knoll, University of Wisconsin (Madison, WI, USA). After four hours of infection, the culture medium was replaced with differentiation medium (high-glucose DMEM powder, 50 mM HEPES, 1% FBS, pH 8.2, penicillin, and streptomycin 1X), and the plates were placed in an incubator at 37 °C in a 0% CO_2_ environment for infection evaluation. The differentiation medium was refreshed daily to minimize the likelihood of the bradyzoites reverting to tachyzoites. For immunofluorescence staining, HFF were fixed with 4% formaldehyde solution and permeabilized using 100 μL of 0.2% Triton X-100 in PBS 1X for 10 min. The cells were incubated overnight at 4 °C in a humidified chamber with constant shaking in the presence of a primary anti-BAG1 monoclonal antibody (1:200 dilution). This antibody was generously provided by Dr. Louis Weiss of the Albert Einstein College of Medicine, University of Bronx, New York, NY, USA. The cells were washed three times with PBS 1X (pH 7.4) for 5 min each, followed by the addition of 100 μL of a 1:5000 solution of FITC-conjugated anti-mouse goat IgG secondary antibody (Abcam ab6785, Cambridge, UK). DAPI (4′,6-Diamidine-2′-phenylindole dihydrochloride) staining solution (reference 10236276001, Sigma Aldrich, USA) was applied at a concentration of 1 μg/mL for one minute to stain the cell nuclei. The round coverslips were carefully detached from the bottom of a 24-well plate and examined at 530 nm for FITC staining and 430 nm for DAPI staining using an Evos FL Auto Cell Imaging System (Life Technologies, USA). The slides were analyzed using zigzag scans to cover the entire surface. During observation, cysts containing bradyzoites were counted using a 40X objective. Tr-H-CHCl3 was added at concentrations of 1, 5, and 10 μg/mL, in triplicate. JAG21, generously provided by Dr. Rima McLeod of the University of Chicago, was previously identified as a potent inhibitor of bradyzoites in vitro [20]. It was used at a concentration of 2 μM as a positive control, DMEM served as a negative control, and 1% dimethyl sulfoxide (DMSO) was used as a vehicle control. The concentrations of DMSO and JAG21 were determined based on McPhillie et al. [20]. The plates were incubated at 37 °C in an atmosphere of 0% CO_2_ for 72 h. Following this incubation period, immunofluorescence was performed, as previously described [21]. The effect of the Tr-H-CHCl3 fraction on cysts was evaluated in both control and treated cultures on the third day. Preliminary analysis revealed that a three-day exposure of 5 × 10^4^ tachyzoites to an alkaline environment coupled with the absence of CO_2_ was the most effective condition for producing bradyzoites (Supplementary Material, Figures S1 and S2). Coverslips were cultured in DMEM alone, with 1% DMSO as a vehicle control, with JAG21 (2 μM), or with 1, 5, or 10 µg/mL of Tr-H-CHCl_3_. Clusters of bradyzoites were quantified three days post-culture. In the present study, we designated the groups of bradyzoites as clusters because there was no cyst wall staining to ensure that the bradyzoite-containing structures were true cysts.

### 2.11. Molecular Docking

The primary candidate targets for the most promising synthetic compound within the thiazolidine group, specifically compound 9, are the Calcium-Dependent Protein Kinase 1, also referred to as TgCDPK1 (TGME49_301440) with PDB code 4JBV, and the ROP18 kinase (TgROP18) with PDB code 4JRN [16]. Molecular docking studies were performed for these two targets. The three-dimensional structure of the compounds was derived by converting the SMILES format using the NovoPro server (www.novoprolabs.com). The receptors (TgCDPK1, TgROP18) and ligands were prepared utilizing Autodock Tools v1.5.7 software (https://autodock.scripps.edu/download-autodock4/ (accessed on 12 November 2025)), where water molecules were removed and Gasteiger charges were calculated. Briefly, the protein receptor was minimized using the YASARA minimization server (https://www.yasara.org/minimizationserver.htm (accessed on 12 November 2025)). The search boxes were customized in terms of dimensions and coordinates according to the protein binding sites of TgCDPK1 and TgROP18, as described by UniProt (https://www.uniprot.org/ (accessed on 12 November 2025)). The PyRx software v0.8 (https://pyrx.sourceforge.io/ (accessed on 12 November 2025)) was employed for the molecular docking procedure, with an exhaustiveness parameter set to 15. Molecular docking of ATP and the protein binding sites of CDPK1 and TgROP18 with ATP was performed as a reference control. The interactions between promising ligands (one per group) and proteins were identified using Biovia Discovery Studio (https://discover.3ds.com/discovery-studio-visualizer-download (accessed on (accessed on 12 November 2025)) and Chimera software (https://www.cgl.ucsf.edu/chimera/ (accessed on accessed on 12 December 2025)).

## 3. Results

### 3.1. Cytotoxicity, Anti-Toxoplasma Inhibitory Effect and Selectivity Index of the Synthetic and Natural Compounds

The results of the cytotoxicity test (CC50), anti-*Toxoplasma* inhibitory effect (IC50), and selectivity index (SI) for the 72 h continuous exposure screening, as well as the pretreatment assays of host cells and parasites involving the synthetic molecules (1–16) and *T. rosae* fraction, are presented in Table 1. A CC50 value exceeding 160 µM indicates low cytotoxicity for host cells, while an IC50 value exceeding 10 μM suggests compounds with reduced antiparasitic activity in vitro, rendering them less promising for further experimental analysis [6]. Furthermore, an SI value > 50 suggests a safe margin between cytotoxicity and anti-*Toxoplasma* inhibitory activity. We analyzed the pharmacological properties of various compound groups, focusing on in vitro results. Among the pyrazoline compounds, three (1, 2, and 4) exhibited IC50 values below 10 µM. However, upon calculating the selectivity index (SI), only compound 2 exhibited an SI greater than 50. All pyrazole derivatives displayed lower toxicity levels (>160 µM) and high IC50 values, precluding further analysis. Most thiazolidinone derivatives exhibited low IC50 values, indicating significant anti-*Toxoplasma* activity, although some had lower CC50 values, and all had SI values of less than 50. When evaluating the results of host cell pretreatment, the IC50 values were consistently higher than those obtained from continuous exposure, suggesting that the effect was not mediated through host cell targets. Conversely, three thiazolidinone compounds (9, 10, and 15) exhibited lower IC50 values than those observed during 72 h continuous exposure, indicating a direct effect on the parasite targets of these compounds. Notably, compound 9 demonstrated a significant reduction in IC50 (0.9 μM) when the tachyzoites were pre-incubated, suggesting a substantial direct effect on the viability of extracellular *T. gondii* tachyzoites. The *T. rosae* and *T. chrysantha* chloroform fractions confirmed a modest IC50 (6.8 and 7.3, respectively) relative to the synthetic compounds, indicating the need to identify and purify the compounds from these fractions to explain the inhibitory effect on *Toxoplasma* tachyzoites.

### 3.2. In Silico Prediction of Pharmacokinetics, Drug-Likeliness, and ADMET Depiction Exploration

Table 2 shows the outcomes of the *in silico* prediction of the pharmacokinetic properties of the compounds. We included β-amyrin due to its identification as the component responsible for the anti-Toxoplasma activity of *T. rosae* leaves [9]. Regarding the physicochemical properties assessed using pkCSM and in accordance with Lipinski’s rules—specifically, molecular weight (MW) ≤ 500, Log P (octanol/water) > 5, hydrogen bond acceptors ≤ 10, and hydrogen bond donors > 5—only four out of 16 compounds, particularly those with pyrazole nuclei, violated one of Lipinski’s rules by exhibiting a Log P (octanol/water) greater than 5.

In terms of pharmacokinetic properties (Table 3), pyrazole and pyrazoline derivatives are likely to exhibit good intestinal absorption, with human intestinal absorption (HIA) exceeding 90%. However, the thiazolidinone derivatives demonstrated variability in their absorption values; for example, compounds 9, 13, 15, and 17 exhibited absorption values ranging from 70% to 80%. Notably, a compound may be considered poorly absorbed if its absorption value is less than 30%. However, for the volume of distribution (Log L/Kg), the majority of the thiazolidinone derivative compounds were predicted to be poorly distributed (>0.15), and only one pyrazoline derivative compound (2) had a high value (>0.45). For the Blood–Brain Barrier (BBB), the values ranged from −1.181 to 0.272; thiazolidinone compounds 13, 15, and 16 had poor distribution to the brain according to the logBBB (<−1), although none of them could readily cross the blood–brain barrier (>0.3). However, CNS (central nervous system) permeability with the blood-brain permeability-surface area (logPS) is a more direct measurement, with this consideration, the compounds 2, 5, 6, 7, and 8 are considered to penetrate the central nervous system. None of the compounds were CYP2D6 substrates (CYP: Cytochrome P450); however, all pyrazoline and pyrazole compounds and thiazolidinone derivative compounds 12 and 15 were CYP3A4 substrates. Compounds 1 and 2 were inhibitors of 1A2, 2C19, and 2C9; compound 3 was a 1A2 and 2C19 inhibitor; compound 4 was a 1A2, 2C19, and 3A4 inhibitor; compounds 5, 6, 7, and 8 were 1A2, 2C19, and 3A4 inhibitors; and compound 14 was a 1A2 inhibitor of cytochrome. The compounds exhibited predicted total clearance values (Log ml/min/kg) between −0.152 and 0.452, and compounds 1, 10, 12, 14, 15, and 16 were predicted as non-mutagenic.

### 3.3. Result of Anti-Bradyzoite Evaluation of T. rosae Fraction

We selected the chloroform fraction with the lowest IC (Tr-HCHCl3) to determine whether it possessed anti-bradyzoite activity. In Section 2.5, we indicated that infected cells without treatment (IC) were used; accordingly, the extract with the lowest IC was the one with the highest anti-toxoplasma activity and was therefore used for the anti-bradyzoite assay. The exposure of culture to the vehicle DMSO 1% alone reduced significantly the number of cysts containing bradyzoites, all subsequent comparisons for the effect of JAG21 or Tr-H-CHCl_3_ fraction were made with the vehicle. The percentage reduction in the number of bradyzoite clusters was significant compared to the vehicle control for JAG21 (Table 4). For the different concentrations of Tr-H-CHCl_3_ (1 or 5 or 10 µg/mL) the reduction was significantly higher than JAG21, reaching a maximum of 66% at the maximum concentration, exceeding the effect observed with JAG21.

**Figure 1:**
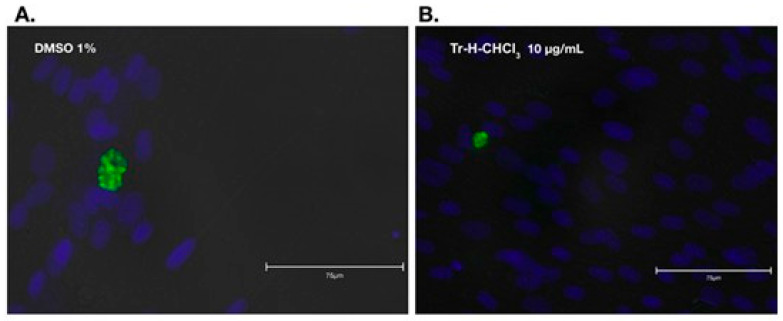
(**A**) Bradyzoites cultured in DMEM containing vehicle control (1% DMSO) after three days. The cluster had a typical size of approximately 30 μm, and more than 16 bradyzoites could be counted. (**B**) Clusters of bradyzoites cultured in DMEM with 10 μg/mL Tr-H-CHCl3 fraction after three days. The cluster is approximately 10 μm long, and seven bradyzoites can be counted inside. Bradyzoites were detected by staining with an anti-BAG1 monoclonal antibody (green) and cell nuclei were visualized with DAPI (blue). Images were obtained by fluorescence microscopy with a 40× objective on an Evos 5000 Microscope after merging visualizations from the 530 nm and 430 nm filters.

Bradyzoites exhibited less dense clustering and were smaller in size, with a reduced number of bradyzoites, compared with those exposed to the 1% DMSO vehicle control (Figure 1).

### 3.4. Molecular Docking of Thiazolidinone Compound 9 with Target Proteins Candidates of T. gondii

**Figure 2:**
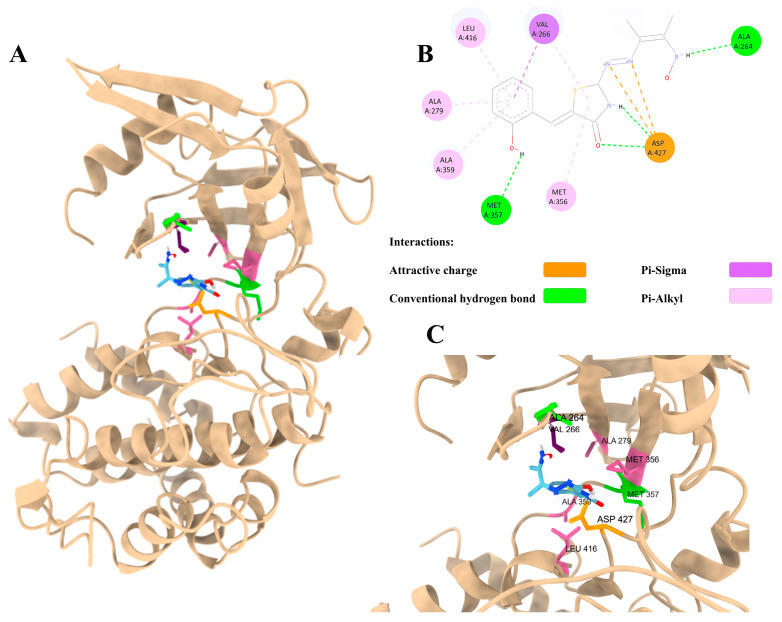
Predicted binding modes of ligand 9 in TgROP18. (**A**) General view of the binding mode of ligand 9 to *Tg*ROP18. Interactions between ligand 9 in 2D (**B**) and 3D (**C**) diagrams with the *Tg*ROP18 protein.

The results of the molecular docking studies, as presented in Table 5, indicate that ligand number 9 from the thiazolidinone group exhibits comparable affinity energies for the TgCDPK1 protein and TgROP18, with values of −6.6 and −8.0 kcal/mol, respectively. The molecular docking of both proteins with their natural ligand, ATP, yields affinity energies of −7.3 for TgCDPK1 and −7.4 for TgROP18. In the case of TgROP18, ligand 9 engages in multiple stabilizing interactions a combination of electrostatic, hydrogen-bond, and hydrophobic contacts. Key polar interactions include conventional hydrogen bonds with ALA(A:264) and MET(A:357), as well as attractive charge interactions with ASP(A:427), which plays a central role in anchoring the ligand. Hydrophobic stabilization is contributed by several π-alkyl contacts with ALA(A:279), ALA(A:359), MET(A:356), and LEU(A:416), together with a π–sigma interaction with VAL(A:266) that supports the positioning of the aromatic ring. Overall, these interactions collectively promote a stable binding pose of the ligand within the active site (Figure 2). 

## 4. Discussion

Our results, which show distinct inhibitory profiles for synthetic (chemically defined) versus natural (plant-derived) agents, underline the value of integrated screening strategies. Synthetic compounds typically offer more uniform performance characteristics (e.g., reproducible activity, known molecular structure, and defined purity), which emerged in our assays as relatively consistent inhibition of *T. gondii* tachyzoite proliferation. For example, previous work has shown that a series of thiosemicarbazides, 4-thiazolidinones and 1,3,4-thiadiazoles display excellent anti-*T. gondii* activity in vitro, compared to standard agents such as sulfadiazine [8,16,22]. In this study, we identified an additional compound, designated as number 9, which exhibited significant activity against extracellular parasites. The potential targets for these compounds have been identified as ROP18 or TgCPK1 protein kinases, which are essential for the proliferation of rapidly dividing tachyzoites [16,23,24]. Docking analysis revealed that the thiazolidinone derivative effectively occupied the active sites of TgROP18 and was stabilized through a combination of hydrogen bonding, π-type interactions, and electrostatic contacts. These interactions likely contribute to their potential inhibitory activity.

In contrast, the natural compound arm of our screening revealed a broader spectrum of activity, from modest action on tachyzoites to unexpectedly strong inhibition of *T. gondii* bradyzoite proliferation. This variability was observed in the previous evaluation of *Tabebuia* extracts [9]. The inherent complexity of plant extracts and their secondary metabolites may act in synergy or antagonism, and batch-to-batch variation is often significant [25]. The detection of BAG1 with a monoclonal antibody in the culture confirmed that the parasites had made the transitioned from tachyzoites to bradyzoites, thus validating the effectiveness of the experimental conditions used in this study. In addition, the average size of the observed clusters was consistent with that reported in the literature. The chloroform fraction obtained from *T. rosea* leaves showed promising activity against the bradyzoite stage. At a concentration of 10 μg/mL, this fraction reduced bradyzoites clusters by 70%, even exceeding the effect of the positive control compound JAG21, known for its action on bradyzoites [20]. The effect observed with the chloroform fraction of *T. rosea* was consistent with studies on chloroform fractions of other plants that have demonstrated antiparasitic activity [26]. These findings suggest that the metabolites present in plant chloroform fractions may interfere with parasite survival, replication, or differentiation. Previous studies have shown that the chloroform extract of *Tabebuia ochracea* ssp. *neochrysanta* exhibits antimalarial activity in vitro against strains of *Plasmodium berghei*, a protozoan that causes malaria in rodents, owing to the presence of furanonaftoquinones [26]. Since *T. rosea* belongs to the same genus, it is possible that it shares similar bioactive compounds, such as naphthoquinones, phenols, and flavonoids, which could affect the viability and stability of *T. gondii* bradyzoites. The chloroform fraction contained β-amyrine, a pentacyclic triterpene isolated from the chloroformic extract of leaves of this species [9]. The present results reinforce the potential of the compounds present in *T. rosea* not only to inhibit the proliferation of tachyzoites but also to impact the more resistant stages of the life cycle of *T. gondii*, such as the bradyzoites in this study. The diverse bioactive properties observed suggest a potential synergistic interaction among the various compounds present, thereby enhancing its therapeutic efficacy in treating parasitic infections such as toxoplasmosis in the chronic phase. This stage is notably challenging to address because of the parasite’s resistance to conventional pharmacological treatments. Further studies are necessary to elucidate the underlying mechanisms. One mechanism of action of natural triterpenes, such as beta-amyrin, is an active area of research. Based on related studies on similar compounds, probable targets or pathways may include calcium-dependent protein kinases (CDPKs). Other lupane-type triterpenes (like betulone derivatives) have been suggested to target *T. gondii* CDPK3 via reverse docking studies [27]. It is possible that beta-amyrin affects a related signaling pathway.

The in silico pharmacokinetic predictions provided a preliminary assessment of the drug-likeliness and ADMET (Absorption, Distribution, Metabolism, Excretion, Toxicity) profiles of the synthetic compounds. Most compounds adhered to Lipinski’s rules and showed good oral bioavailability. Pyrazole compounds exhibit good intestinal absorption and can penetrate the central nervous system (CNS). CNS penetration is particularly relevant for treating the chronic stage of toxoplasmosis, where parasites reside within brain tissue cysts [1]. Compound 2, with its high volume of distribution and predicted CNS permeability, is a particularly interesting candidate for further in vivo studies.

Metabolic profiling has indicated that many compounds are substrates for CYP3A4, which could lead to drug–drug interactions if co-administered with other medications metabolized by this pathway. This information is crucial for guiding future clinical developments. Toxicity predictions have highlighted several non-mutagenic candidates (1, 10, 12, 14, 15, 16), which is a positive indicator of safety.

For labs in endemic and often under-resourced regions, the search for anti-parasitic compounds has two major implications: (1) it shows that local biodiversity can be leveraged even without access to large synthetic libraries; and (2) it highlights the need for robust assay standardization (e.g., extraction protocol, parasite strain, host cell line, exposure time) to reduce variability and improve comparability. Indeed, the literature emphasizes this variability, differences in extraction method, solvent, plant part and parasite strain likely account for divergent results among anti-*T. gondii* herbal-extract studies [25].

Therefore, our concurrent assessment of synthetic and natural compounds yielded several strategic insights.

- Benchmarking and controls: Synthetic compounds serve as reliable internal standards, helping to measure assay drift and enabling cross-comparisons among experiments.
- Discovery funneling: Plant extracts with promising activity may feed into synthetic-lead optimization pipelines, combining the novelty of natural chemistry with the reproducibility of synthetic optimization.
- Resistance mitigation: Because natural compounds may act via unconventional or multiple targets, they might provide a way to circumvent or delay the resistance pathways that plague synthetic drugs.

In endemic country settings, resource constraints (reagents, equipment maintenance, strain availability, and infrastructure) impose additional experimental variability and drive innovation. From our workflow, we highlight the following points:

- Parallel screening design: Running synthetic and natural compound arms side-by-side can help normalize inter-run variability and provide cross-validation of assay performance.
- Local extraction and standardization: Standardized protocols for crude natural-product extraction (solvent, plant part, and dose metrics) are essential; heterogeneity in methodology significantly degrades comparability.
- Diverse strain panel: Because *T. gondii* genotypic diversity in endemic settings (especially in Latin America) is high, assays should consider testing across multiple strains to capture potential variation in susceptibility [28].

From a broader perspective, our findings emphasize that meaningful advances in antiparasitic research are possible even under constrained settings, provided that the experimental design is rigorous and context relevant. Harnessing regional biodiversity via natural-product screening complements global synthetic-compound efforts and could lead to locally relevant therapeutic leads (e.g., compounds derived from endemic plants or fungi). Moreover, combining natural and synthetic pipelines may enhance the global armamentarium against *T. gondii* by diversifying the mechanisms of action and reducing the risk of cross-resistance.

In the specific context of toxoplasmosis (which remains a significant burden in many tropical countries), the fact that both synthetic and natural agents show measurable in vitro efficacy argues for a two-pronged approach: continued refinement of synthetic candidate libraries while simultaneously expanding natural-product screening efforts. When considering translation to clinical or in vivo settings, regional capacity for toxicity testing, formulation, pharmacokinetics, and field-relevant parasite strains must also be considered.

Our in vitro model, while valuable, cannot fully replicate in vivo complexities (host immunity, tissue distribution, bradyzoite stage, and latent cysts). In future research, it is imperative to conduct experiments utilizing the ME49 Pru strain, a cystogenic lineage [19], to further assess promising compounds such as thiazolidinone 9. These investigations should encompass assays evaluating parasite viability during the tachyzoite stage and should also extend to the cyst stage. Many natural compounds lack mechanistic annotation, which hinders rational optimization. Resistance to synthetic leads remains a significant issue. Future work should ideally include time-kill and combination studies (synthetic and natural) to assess synergistic or additive effects; in vivo efficacy testing (including chronic infection models and cyst burden) in relevant host systems; mechanistic studies to elucidate molecular targets, especially for active natural compounds; strain-diversity testing to ensure broad efficacy across genotypes found in endemic regions; and development of standardized protocols and reagent sharing networks among endemic-country labs to boost comparability and capacity.

## 5. Conclusions

In summary, our concurrent assessment of synthetic and natural compounds against *T. gondii* proliferation highlights both the potential and practical challenges of antiparasitic research in tropical settings. Synthetic compounds provide reproducible benchmarks and potential drug leads, whereas natural compounds provide chemical novelty and context-relevant opportunities. Our findings justify further in vivo evaluation of the most promising synthetic candidates to assess their efficacy in eradicating both acute and chronic *T. gondii* infection. When deployed together in an integrated screening framework supported by methodical design and local capacity, this dual strategy holds significant potential for advancing toxoplasmosis research and drug discovery efforts in endemic countries.

## Figures and Tables

**Table 1:** Results of half-maximal cytotoxic concentration (CC50) on Vero cells, the anti-*Toxoplasma* half-maximal inhibitory concentration (IC50), and the selectivity index (SI) ratio of CC50 to IC50 for pyrazoline, pyrazole, and thiazolidinone synthetic compounds, as well as the *T. rosae* chloroform fraction from leaves. CC50 was determined using the Alamar blue technique, whereas IC50 was assessed through GFP fluorescent tachyzoite viability quantification.

					Host Cell Pre-Treatment		Tachyzoite Pre-Treatment	
Number of the Compound	Group	CC_50_ (µM)	IC_50_ (µM)	SI	IC_50_ (µM)	SI	IC_50_ (µM)	SI
1	Pyrazoline	131.9	6.7	19.6	7.5	17.5	23.4	5.6
2		123.3	1.6	73.3	7.5	16.4	6.6	18.6
3		>160	18.1	>8.3	-	-	-	-
4		154.2	3.6	42.6	5.3	28.9	31.6	4.8
5	Pyrazole	>160	14.0	>11.4	-	-	-	-
6		>160	21.3	>7.5	-	-	-	-
7		>160	15.9	>10.0	-	-	-	-
8		>160	21.0	>7.6	-	-	-	-
9	Thiazolidinone	>160	6.1	>25.9	10.7	>14.9	0.9	>164.9
10		61.2	5.7	10.6	15.4	3.9	4.9	12.4
11		53.2	3.6	14.5	22.5	2.3	29.5	1.8
12		40.3	3.9	10.2	10.5	3.8	5.8	6.9
13		>160	8.8	>18.1	254.6	>0.6	19.8	>8.0
14		>160	141.6	>1.1	-	-	-	-
15		>160	9.5	>16.8	54.4	>2.9	4.0	>39.7
16		>160	16.7	>9.5	-	-	-	-
Tr-HCHCl3	Plant extract	50.1	6.8	7.3	-	-	-	-
Tc-HCHCL3	Plant extract	24	5.6	4.4				
Pyrimethamine		>200	0.3	>666	1.0	>190	6.1	>32.4

**Table 2:** Prediction of physico-chemical parameters of pyrazoline, pyrazole and thiazolidinone derivatives ligands based on Lipinski regulations.

Ligand	Group	Physical and Chemical Properties					
		Molecular Weight (g/mol)	Rotatable Bonds Number	Log P (Octanol/ Water)	H-Bond Acceptors Number	H-Bond Donors Number	Lipinski Violations
1	Pyrazoline	286.30	3	2.97	4	2	0/5
2		302.76	3	3.49	4	2	0/5
3		298.34	4	2.84	5	2	0/5
4		312.32	3	2.56	6	2	0/5
5	Pyrazole	409.32	3	6.63	4	1	1/5
6		374.87	3	5.97	4	1	1/5
7		358.42	3	5.46	4	1	1/5
8		419.32	3	6.08	4	1	1/5
9	Thiazolidinone	318.35	3	2.17	7	3	0/5
10		318.35	3	2.17	7	3	0/5
11		327.36	3	2.34	7	2	0/5
12		332.38	4	2.48	7	2	0/5
13		334.35	3	1.88	8	4	0/5
14		336.80	3	3.12	6	2	0/5
15		369.40	3	2.31	8	3	0/5
16		284.297	4	0.488	8	2	0/5
	β-amyrin	396.69	0	8.66	0	0	1/5

**Table 3:** ADMET-Tox pharmacokinetic predicted properties of pyrazoline, pyrazole and thiazolidinone derivatives ligands.

		Absorption	Distribution			Metabolism							Excretion	Toxicity
Ligand	Group	Intestinal absorption (human)	VDss (human)	BBB permeability	CNS permeability	Substrate		Inhibitor					Total Clearance	AMES toxicity
						CYP								
						2D6	3A4	1A2	2C19	2C9	2D6	3A4		
		Numeric (% Absorbed)	Numeric (Log L/kg)	Numeric (Log BBB)	Numeric (Log PS)	Categorical (Yes/No)							Numeric (Log mL/min/kg)	Categorical (Yes/No)
1	Pyrazoline	92.349	0.390	0.265	−2.131	No	Yes	Yes	Yes	Yes	No	No	0.183	No
2		91.392	0.478	0.272	−1.968	No	Yes	Yes	Yes	Yes	No	No	0.133	Yes
3		93.901	0.260	0.022	−2.245	No	Yes	Yes	Yes	No	No	No	0.32	Yes
4		93.218	0.369	−0.137	−2.322	No	Yes	Yes	Yes	No	No	Yes	0.17	Yes
5	Pyrazole	90.699	0.078	0.22	−1.087	No	Yes	Yes	Yes	Yes	No	Yes	0.34	Yes
6		92.074	0.013	0.193	−1.202	No	Yes	Yes	Yes	Yes	No	Yes	0.221	Yes
7		93.142	−0.099	0.066	−1.365	No	Yes	Yes	Yes	Yes	No	Yes	0.452	Yes
8		92.007	0.03	0.191	−1.18	No	Yes	Yes	Yes	Yes	No	Yes	0.199	Yes
9	Thiazolidinone	75.508	−0.177	−0.919	−2.483	No	No	No	No	No	No	No	0.068	Yes
10		80.849	−0.306	−0.945	−2.494	No	No	No	No	No	No	No	−0.052	No
11		81.681	−0.358	−0.537	−2.365	No	No	No	No	No	No	No	0.03	Yes
12		82.429	−0.304	−0.774	−2.506	No	Yes	No	No	No	No	No	0.097	No
13		70.116	−0.166	−1.181	−3.48	No	No	No	No	No	No	No	0.054	Yes
14		90.208	−0.26	−0.609	−2.189	No	No	Yes	No	No	No	No	−0.067	No
15		78.711	−0.132	−1.019	−3.038	No	Yes	No	No	No	No	No	−0.152	No
16		70.155	−0.601	−1.008	−3.181	No	No	No	No	No	No	No	0.276	No
β Amyrin		100.000	-	0.97	−1.277	Yes	Yes	No	No	Yes	Yes	Yes	-	No

**Table 4:** The effect on the mean ± SD number of *T. gondii* of clusters containing bradyzoites. The means were derived from triplicates in two independent experiments (*n* = 6). Statistically significant *p*-values determined using a non-parametric *t*-test are indicated in bold (*p* < 0.05).

Condition	DMEM Alone	DMSO 1%	JAG21 (2 µM)	Tr-H-CHCl_3_ 1 µg/mL	Tr-H-CHCl_3_ 5 µg/mL	Tr-H-CHCl_3_ 10 µg/mL
Mean number of cysts in 10 fields	52 ± 5.4	38 ± 2.8	26.67 ± 5.8	21.1 ± 2.2	19.3 ± 3.0	12.8 ± 3.3
% reduction vs. DMEM	-	27.2	48.9	59.4	62.9	75.4
*p* vs. DMEM	-	0.00065	0.000015	0.0000066	0.0000017	0.0000003
% reduction vs. DMSO	**-**	-	29.8	44.3	49.1	66.2
*p* vs. DMSO	**-**	-	0.01	0.00039	0.00013677	1.2005 × 10^−5^
% reduction vs. JAG21	**-**	-	-	20.6	34.6	71.6
*p* vs. JAG21	**-**	-	-	0.073	0.028	0.001

**Table 5:** Binding affinity scores in kcal/mol between ligand 9 and proteins *Tg*CDPK1 and *Tg*ROP18.

Ligand	Group	Affinity *Tg*CDPK1 (kcal/mol)	Affinity *Tg*ROP18 (kcal/mol)
9	Thiazolidinone	−6.6	−8.0
ATP	Control	−7.3	−7.4

## Data Availability

The original contributions presented in this study are included in the article/Supplementary Material. Further inquiries can be directed to the corresponding author.

## Supplementary Materials

The following supporting information can be downloaded at: https://www.mdpi.com/article/10.3390/tropicalmed10120349/s1, Scheme S1: General synthesis of derivatives 1–4; Scheme S2: General synthesis of derivatives 5–8; Scheme S3: General synthesis of derivatives 9–16; Supplementary Table S1. Phytochemical analysis of extracts obtained from Tabebuia rosea leaves. Supplementary Table S2. Phytochemical analysis of extracts obtained from Tabebuia rosea inner bark; Supplementary Table S3. Phytochemical analysis of extracts obtained from Tabebuia chrysantha inner bark; Supplementary Table S4. Phytochemical analysis of extracts obtained from Tabebuia chrysantha leaves; Supplementary Figure S1: Immunofluorescence assays with anti BAG1; Supplementary Figure S2: Experiments of obtention of bradyzoites in vitro. Supplementary Figure S3: FTIR compound 9; Supplementary Figure S4: FTIR compound 10; Supplementary Figure S5: FTIR compound 11; Supplementary Figure S6: FTIR compound 12; Supplementary Figure S7: FTIR compound 13; Supplementary Figure S8: FTIR compound 14; Supplementary Figure S9: ^1^H NMR compound 15; Supplementary Figure S10: ^13^C NMR compound 15; Supplementary Figure S11: FTIR compound 15.
