# Supplementary material for: Concurrent Assessment of Synthetic and Natural Compounds on the Proliferation of Toxoplasma gondii in In Vitro Models
Source: Trop Med Infect Dis. 2025 Dec 13;10(12):349. doi: 10.3390/tropicalmed10120349 (PMC12737779; doi:10.3390/tropicalmed10120349)
Supplement: Supplementary file 1 [file tropicalmed-10-00349-s001.zip › tropicalmed-4018483-supplementary.pdf]

Supplementary material

1. **Synthesis of compounds**

1.1 2-Pyrazoline synthesis

A chalcone (β-unsaturated ketone) derivative (**1-4**) was synthesized via a conventional base-catalyzed Claisen–Schmidt condensation reaction. The aldehyde and ketone were dissolved in absolute ethanol and NaOH, as depicted in scheme 3. This allowed the formation of the α, β-unsaturated compound, which was then treated with hydrazine hydrate and heated to reflux. This intramolecular cyclization reaction facilitated the formation of 2-pyrazolines, in which vanillin represents the A-ring, while aryl substituted represents the B-ring (**Scheme 1**).

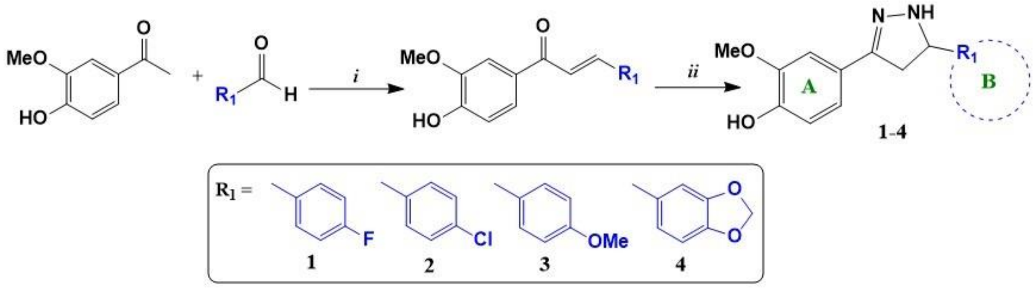

Scheme S1: General synthesis of derivatives **1-4.** Reagents and conditions: (*i*) NaOH 20%, EtOH, r.t. overnight; (*ii*) Ethanol, hydrazine hydrate, reflux, 3h.

1.2 (Indol-3-yl)-pyrazole-4-carbonitrile synthesis

General procedure for the synthesis of compound 5-8

The general synthesis of 1H-indole-1H-pyrazol-4-carbonitrile derivatives **5-8** (**scheme 2**) begins with the cyanoacetylation of indole by the reaction between indole (0.11g, 1mmol) and cyanoacetic acid (0.0085 g, 1 mmol) mediated by acetic anhydride (5 mL) to obtain of 3-cyanoacetylindole **d** isolated as solid product by simple filtration [23]. The Knoevenagel condensation of the compound methylene activated **d**, with aromatic aldehydes in the ethanolic triethylamine under reflux conditions, furnished the corresponding acrylamide derivatives **e** in excellent yield [24]. Finally, the indole-pyrazole **5-8** was obtained by reaction between chalcone intermediate **d** and tert-butyl hydrazine in the presence of triethylamine under refluxing heating [25].

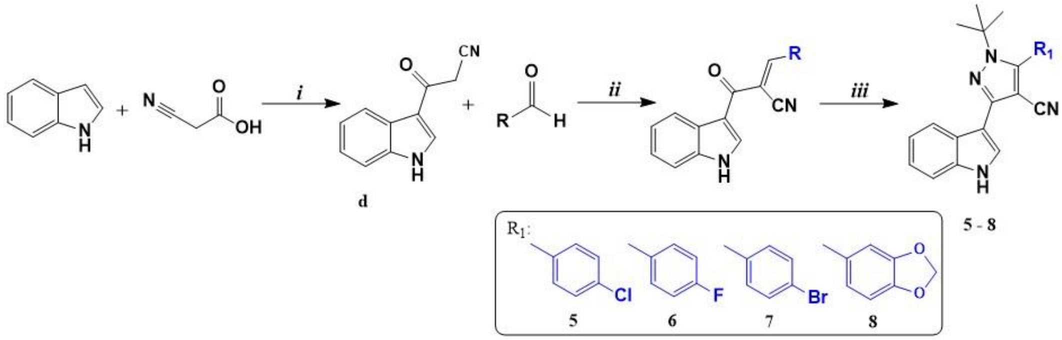

Scheme S2: General synthesis of derivatives **5-8.** Reagents and conditions: (*i*) Acetic anhydride and cyanoacetic acid at 50 °C for 5 min at 80 °C for 10 min; (*ii*) Ethanol, piperidine, reflux, 1h; (*iii*) Ethanol, tert-butyl hydrazine, reflux, 3h.

1.3 Thiazolidin-4-one synthesis.

General procedure for the synthesis of compound 9-16

The general synthesis of the thiazolidine-4-one derivatives is outlined in **scheme 3**. The diacetyl monoxime reacted directly with thiosemicarbazide in ethanol using acetic acid as the catalyst [20]. The resulting thiosemicarbazones (**a**) reacted with ethyl bromoacetate in methanol and sodium acetate carried out by intramolecular cyclization to obtain 3-hydroxyimino butan-ylidene-2-hydrazonothiazolidin-4-one (**b**)[21]. Finally, the Knoevenagel condensation reaction between aromatic aldehydes and compound (**b**) in the presence of a solution of piperidine with absolute ethanol and reflux heating allows the formation of a yellow solid with a yield of 85-97%, corresponding to the 5-arylidene derivative (**9-16**) [22].

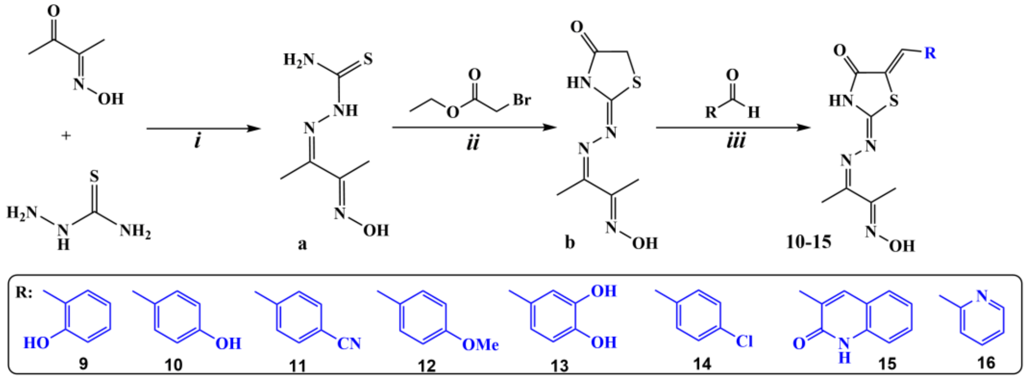

Scheme S3: General synthesis of derivatives **9-16**. Reagents and conditions: (i) Ethanol, acetic acid (cat), reflux, 2 h, yield 75%; (ii) Methanol, CH_3COONa, reflux, 3 h, yield 81%; (iii) Ethanol, piperidine, reflux 2 h, yield 85-97%.

Spectral data

(E)-5-((Z)-2-hydroxybenzylidene)-2-(((2Z,3E)-3-(hydroxyimino) butan-2-ylidene) hydrazono) thiazolidin-4-one (9). Yellow solid, yield = 87%; m.p. 224-226 °C, FTIR (cm^{-1}): 3438 (OH phenol), 3266(NH amide), 3184 (C-H aromatic), 3057-2926-2878 (C-H aliphatic), 1691 (C=O amide), 1618 (C=N imina), 1581 (C=C aromatic), 1241 (C-O phenol), 1129 (N-O hydroxylamine), 1009-522 (C-S thiazole ring).

(E) -5- ((Z)-4-hydroxybenzylidene) -2- (((2Z,3Z)-3-(hydroxyimino) butan-2-ylidene) hydrazono) thiazolidin-4-one (10). Yellow solid; yield = 92%; m. p. 244-246°C; FTIR (cm^{-1}): 3438 (OH phenol), 3272 (NH amide), 3065 (C-H aromatic), 2976-2926-2864 (C-H aliphatic), 1719 (C=O amide), 1614 (C=N imina), 1581 (C=C aromatic), 1241 (C-O phenol), 1205 (C-N oxime), 506 (C-S thiazole ring).

4-((Z)-((E)-2-(((2Z,3Z)-3-(hydroxyimino) butan-2-ylidene) hydrazono)-4-oxothiazolidin-5-ylidene) methyl) benzonitrile (11). Yellow solid; yield = 85%; m. p. 249-251°C; FTIR (cm^{-1}): 3391 (OH oxime), 3264 (NH amide), 3062 (C-H aromatic), 2932-2875 (C-H aliphatic), 2226 (C≡N), 1692 (C=O amide), 1624 (C=N imina), 1606 (C=C aromatic), 1240 (C-N oxime), 1004 (C-S thiazole ring).

(E)-2-(((2Z,3Z)-3-(hydroxyimino) butan-2-ylidene) hydrazono)-5-((Z)-4-methoxybenzylidene) thiazolidin-4-one (12). Yellow solid; yield= 87%; m.p 270-272 °C; FTIR (cm^{-1}): 3424 (OH oxime), 3202 (NH amide), 3090 (C-H aromatic), 3046-3005-2959 (C-H aliphatic), 1689 (C=O amide), 1624 (C=N imina), 1593 (C=C aromatic), 1250 (C-O-C Ether), 1006-503 (C-S thiazole ring).

(E) -5- ((Z)-3,4-dihydroxybenzylidene) -2- (((2Z,3Z)-3-(hydroxyimino) butan-2-ylidene) hydrazono) thiazolidin-4-one (13). Brown solid; yield = 92%; m.p 243-244 °C; FTIR (cm^{-1}): 3427 (OH phenol), 3275 (NH amide), 3070 (C-H aromatic), 2976-2924-2872 (C-H aliphatic), 1720 (C=O amide), 1615 (C=N imina), 1580 (C=C aromatic), 1241 (C-O phenol), 1204 (C-N oxime), 1009-507 (C-S thiazole ring).

(E)-5-((Z)-4-chlorobenzylidene)-2-(((2Z,3Z)-3-(hydroxyimino) butan-2-ylidene) hydrazono) thiazolidin-4-one (14). Orange solid, yield = 97%; m. p. 204-206 °C; FTIR (cm^{-1}): 3267 (NH amide), 3069 (C-H aromatic), 2979-2952 (C-H aliphatic), 1692 (C=O amide), 1624 (C=N imina), 1605 (C=C aromatic), 1200 (C-N oxime), 1009-513 (C-S thiazole ring), 699 (C-Cl).

(2Z,5Z)-2-(((2Z,3Z)-3-(hydroxyimino) butan-2-ylidene) hydrazono)-5-((2-oxo-1,2-dihydroquinolin-3-yl) methylene) thiazolidin-4-one (15). Yellow solid; yield = 88%; m. p. 297-299 °C; FTIR (cm^{-1}): 1697 (C=O amide), 1624 (C=N), 2948 (C-H), 3180 (C-H aromatic), 3423 (N-H). ^{1}H NMR (δ ppm, DMSO-d6, 600 MHz): 12.64 (s, 1H, OH), 12.13 (s, 1H, NH), 11.80 (s, 1H, NH), 8.13 (s, 1H, -CH), 7.81 (d, 1H, 3J=7.5 Hz, -CH), 7.72 (s, 1H, -CH), 7.56 (m, 1H, 3J = 7.06 Hz, -CH), 7.33 (d, 1H, 3J = 8.3 Hz, -CH), 7.23 (m, 1H, 3J = 7.87 Hz, -CH), 2.21 (s, 3H, -CH3), 2.08 (s, 3H, -CH3). ^{13}C NMR (δ ppm, DMSO-d6, 150 MHz): 9.91 (CH3), 13.56 (CH3), 115.59 (CHAr), 119.48 (Cqua), 122.94 (CHAr), 123.55 (CH), 126.04 (Cqua), 126.41 (Cqua), 129.48 (CHAr), 132.16 (CHAr), 139.10 (CHAr), 140.14 (Cqua), 155.35 (Cqua), 160.70 (Cqua), 161.13 (C=O), 168.20 (C=O). HRMS calculated for $C_{17}H_{15}N_5O_3S$: 369.40; found:369.

I D	Molecular structure	Reaction time	Melting point (°C)	Molecula r weight (g/mol	Yiel d (%)	Color
9	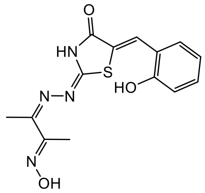	11 h	224-226	318,35	87	Yellow
10	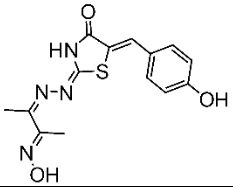	8 h	244 - 246	318,35	92	Yellow
11	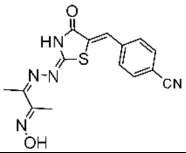	9 h	249 - 251	327,36	85	Yellow
12	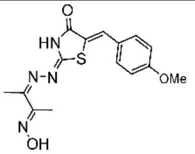	11 h	270 - 272	332,38	87	Yellow
13	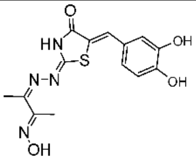	7, 5 h	243 - 244	334,35	92	Brown

14	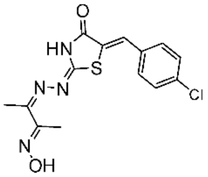	8,<br>5 h	204<br>-<br>206	336,79	97	Orang<br>e
15	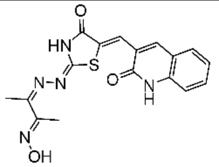	9,<br>5 h	297<br>-<br>299	369,40	88	Yellow

**Structures of compounds**				
Pyrazoline	1	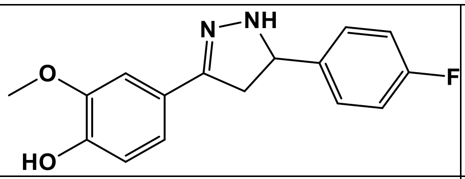	3	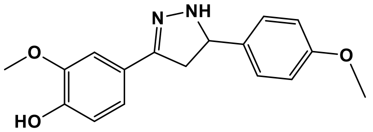
	2	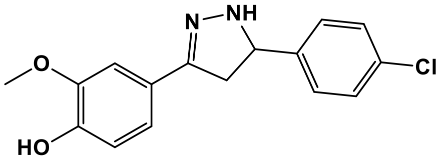	4	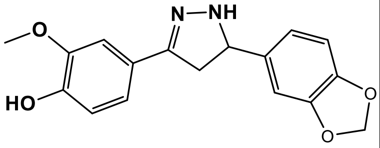
Pyrazole	5	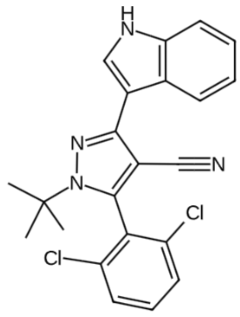	7	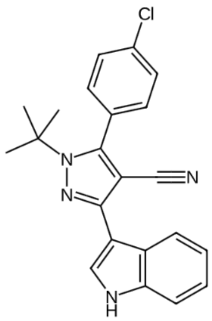
	6	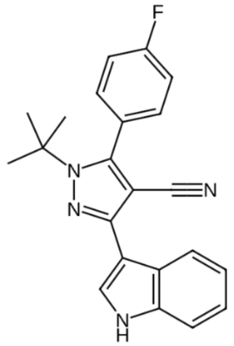	8	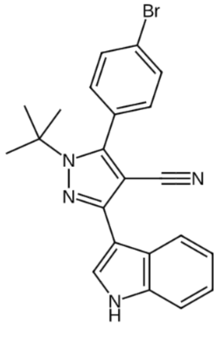

Thiazolidinone	9	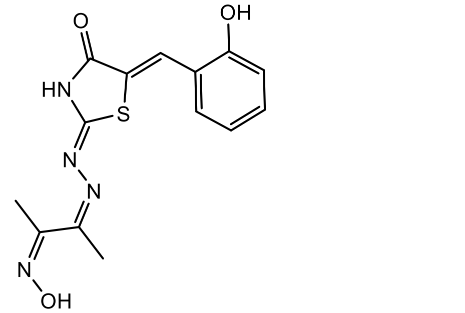	13	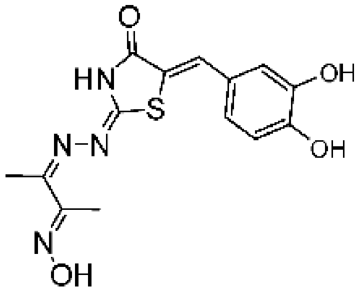
	10	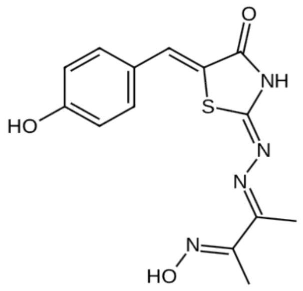	14	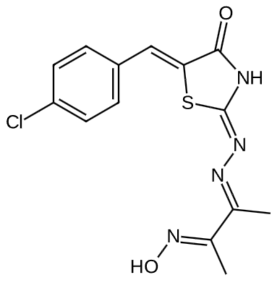
	11	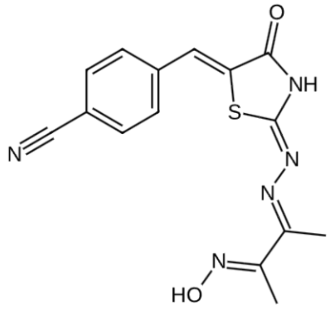	15	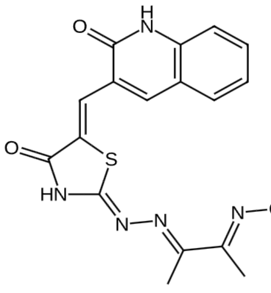
	12	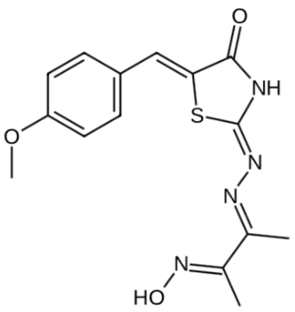	16	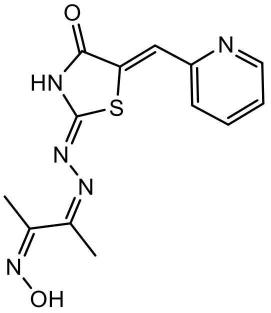

Supplementary Table S1. Phytochemical analysis of extracts obtained from *Tabebuia rosea* leaves.

PHYTOCHEMICAL COMPONENT	REAGENT	MEOH	HEX	$CHCL_3$	ACET (SUN)	ACET (INS)	BUOH	H_2O
PHENOLS AND TANNINS	$FeCL_3$/EtOH	++	-	-	+++	+++	+++	-
FLAVONOIDS	$AlCl_3$/EtOH	++	+	-	+++	+++	++	+
LIGNANS	UV 365 nm	+	-	-	++	++	++	+
	Vanillin/EtOH-H_3PO_4	++	++	++	+++	++	++	++
ANTHRONES	KOH/EtOH	+++	+	++	+++	+++	+++	+
ANTHRAQUINONES		-	-	-	-	-	-	-
COUMARINS		+	+	-	+++	++	++	+
TERPENES/TERPENOIDS STEROLS/STEROIDS	Liebermann-Burchard	+++	+++	+++	+++	++	+++	+
	Vanillin-H_3PO_4	+++	+++	+++	+++	+++	+++	-
IRIDOIDS	Anisaldehyde-AcAc-H_2SO_4	+++	+++	+++	++	-	-	-
	Vanillin-H_2SO_4	+++	+++	+++	+++	-	++	-
TRITERPENES	Anisaldehyde-AcAc-H_2SO_4	++	+++	+++	+	-	-	-
SAPONINS		+++	+++	+++	++	++	++	-
TRITERPENES AND SAPONINS	$SbCl_3$/MeOH	+++	+	++	+++	+++	+++	-
SESQUITERPENE LACTONES	Oleum	-	-	-	-	-	-	-
ALDEHYDES AND KETONES	DNPH	++	+++	++	+++	++	++	+
UNSATURATED FATTY ACIDS	Iodine	++	+++	+++	+++	++	+++	++
ANTIOXIDANT COMPOUNDS	DPPH	+++	+++	+	+++	+++	+++	++

Supplementary Table S2. Phytochemical analysis of extracts obtained from *Tabebuia rosea* inner bark

PHYTOCHEMICAL COMPONENT	REAGENT	MEOH	HEX	$CHCL_3$	ACET (SUN)	ACET (INS)	BUOH	H_2O
**PHENOLS AND TANNINS**	$FeCL_3$/EtOH	+	-	++	+	+	+	-
**FLAVONOIDS**	$AlCl_3$/EtOH	+++	+	+	+++	+++	+++	+
**LIGNANS**	UV 365 nm	++	-	+	++	++	++	+
	Vanillin/EtOH-H_3PO_4	+++	+++	+++	+++	+++	+++	++
**ANTHRONES**		++	-	-	-	++	++	++
**ANTHRAQUINONES**	KOH/EtOH	+	-	+++	-	-	-	-
**COUMARINS**		++	++	+++	+++	+++	+++	+
**TERPENES/TERPENOIDS STEROLS/STEROIDS**	Liebermann-Burchard	++	+++	+++	+++	+++	++	+
	Vanillin-H_3PO_4	+++	++	+++	+++	++	++	++
**IRIDOIDS**	Anisaldehyde- AcAc-H_2SO_4	+	++	++	+	+	-	-
	Vanillin-H_2SO_4	++	+++	+++	+++	++	-	-
**TRITERPENES**	Anisaldehyde- AcAc-H_2SO_4	-	-	-	-	-	-	-
**SAPONINS**		++	+	+++	++	++	++	-
**TRITERPENES AND SAPONINS**	$SbCl_3$/MeOH	++	+++	+++	+++	++	++	-
**SESQUITERPENE LACTONES**	Oleum	-	-	-	-	-	-	-
**ALDEHYDES AND KETONES**	DNPH	++	++	+++	+	++	++	+
**UNSATURATED FATTY ACIDS**	Iodine	+++	+++	+++	+++	+++	+++	++
**ANTIOXIDANT COMPOUNDS**	DPPH	+++	+++	+++	+++	+++	+++	+++

Supplementary Table S3. Phytochemical analysis of extracts obtained from *Tabebuia chrysantha* inner bark

PHYTOCHEMICAL COMPONENT	REAGENT	MEOH	HEX	$CHCL_3$	ACET (SUN)	ACET (INS)	BUOH	H_2O
**PHENOLS AND TANNINS**	$FeCL_3$/EtOH	++	+	+	+++	+++	+++	-
**FLAVONOIDS**	$AlCl_3$/EtOH	++	+	++	+++	+++	+++	-
**LIGNANS**	UV 365 nm	++	-	+	+++	++	++	-
	Vanillin/EtOH-H_3PO_4	+++	+	+	++	++	++	++
**ANTHRONES**	KOH/EtOH	++	+	-	+++	+++	+++	-
**ANTHRAQUINONES**		+	++	+++	-	-	-	-
**COUMARINS**		++	+	++	++	++	++	+
**TERPENES/TERPENOIDS STEROLS/STEROIDS**	Liebermann-Burchard	+++	+++	+++	+++	+++	+++	++
	Vanillin-H_3PO_4	++	+++	+++	+++	+++	+++	++
**IRIDOIDS**	Anisaldehyde-AcAc-H_2SO_4	++	+++	+++	+++	-	-	-
	Vanillin-H_2SO_4	+++	+++	+++	+++	+++	+++	+
**TRITERPENES**	Anisaldehyde-AcAc-H_2SO_4	++	+++	+++	++	-	-	-
**SAPONINS**		++	+++	++	++	-	-	-
**TRITERPENES AND SAPONINS**	$SbCl_3$/MeOH	+	+++	+++	++	++	++	+
**SESQUITERPENE LACTONES**	Oleum	-	-	-	-	-	-	-
**ALDEHYDES AND KETONES**	DNPH	++	+++	+++	++	++	++	-
**UNSATURATED FATTY ACIDS**	Iodine	+++	+++	+++	+++	+++	+++	++
**ANTIOXIDANT COMPOUNDS**	DPPH	+++	+++	+++	+++	+++	+++	+++

Supplementary Table S4. Phytochemical analysis of extracts obtained from *Tabebuia chrysantha* leaves

Phytochemical Core	Reagent	MeOH	CHCl3	AcEt	Buoh	H_2O
Phenols and Tannins	$FeCL_3$/EtOH	++	-	+++	+++	-
Flavonoids	$AlCl_3$/EtOH	+++	-	+++	+++	++
Lignans	UV 365 nm	+++	+	+++	+++	++
	Vanillin/EtOH-H_3PO_4	+++	+	+++	+++	++
Anthrones	KOH/EtOH	++	+	+++	+++	++
Anthraquinones		-	-	+	-	-
Coumarins		+	+	+	++	+
Terpenes/Terpenoids Sterols/Steroids	Liebermann-Burchard	+++	+++	++	++	++
	Vanillin-H_3PO_4	++	+++	+++	+++	++
Iridoids	Anisaldehyde- AcAc-H_2SO_4	+++	+++	++	-	-
	Vanillin-H_2SO_4	+++	+++	+	+	-
Triterpenes	Anisaldehyde- AcAc-H_2SO_4	+++	+++	+	-	-
Saponins		+++	+++	++	++	+
Triterpenes Saponins	$SbCl_3$/MeOH	++	++	++	++	+
Sesquiterpene Lactones	Oleum	-	-	-	-	-
Aldehydes and Ketones	DNPH	+	-	+++	++	++
Unsaturated Fatty Acids	Iodine	+++	+++	+++	+++	+++
Antioxidant compounds	DPPH	+++	++	+++	+++	+++

Supplementary Figure S1. Immunofluorescence assays with anti BAG1

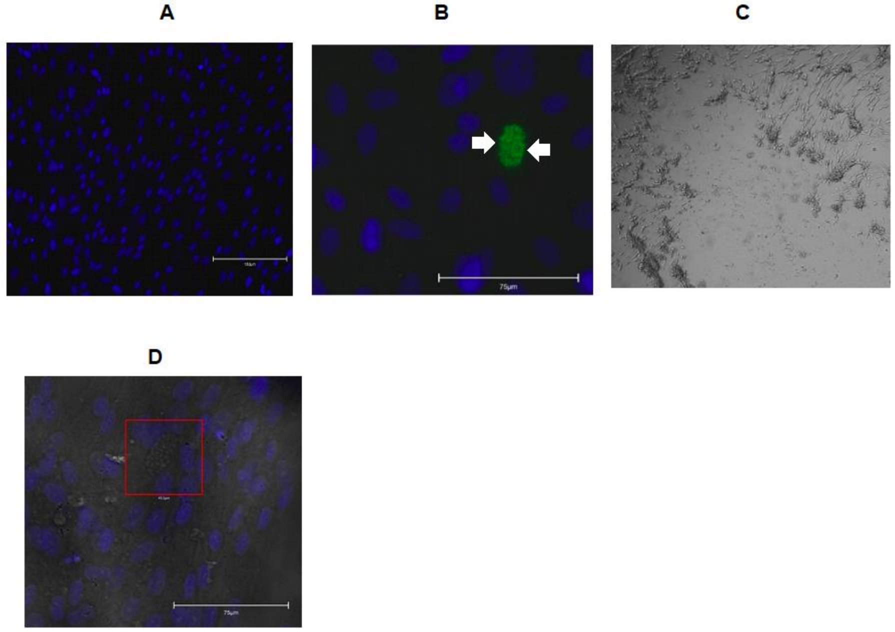

A. An immunofluorescence assay was conducted using an anti-BAG1 antibody and a FITC-labeled secondary antibody for visualization with a 530 nm filter. The assay was performed on a slide inoculated with 5×10^3 PRU tachyzoites on HFF cells under differentiation conditions for three days. No tissue cysts or bradyzoites were observed. Cell nuclei were visualized using a 430 nm filter and stained with DAPI. The scale bar represents 150 mm. The image was captured using a 20X lens on an Evos 5000 Microscope after merging visualizations from the 530 nm and 430 nm filters**. B.** An immunofluorescence assay was conducted using an anti-BAG1 antibody and a FITC-labeled secondary antibody for visualization with a 530 nm filter. The assay was performed on a slide inoculated with 5 x 10^4 PRU strain tachyzoites on HFF cell culture under differentiation conditions for three days. Well-differentiated tissue cysts containing BAG1 green-labeled bradyzoites (indicated by white arrows) within HFF cells were obtained. Scale bar represents 75 m µm. Images were captured using a 40X lens on an Evos 5000 microscope after merging visualizations from the 530 nm and 430 nm filters. **C.** Differential interference contrast image was obtained using an Evos 5000 microscope for an inoculum with 6×10^5 PRU strain tachyzoites under differentiation conditions for three days, demonstrating complete lysis of HFF cells**. D.** A negative control was performed, in which the primary antibody was omitted and replaced with a 3% BSA solution to verify the specificity of the labeling. An infected cell containing unlabeled parasites is indicated by a red square. Scale bar represents 75 m µm. The image was captured using a 40X lens on an Evos 5000 Microscope after merging the visualizations from the 530 nm and 430 nm filters.

Supplementary Figure S2. Experiments of obtention of bradyzoites in vitro.

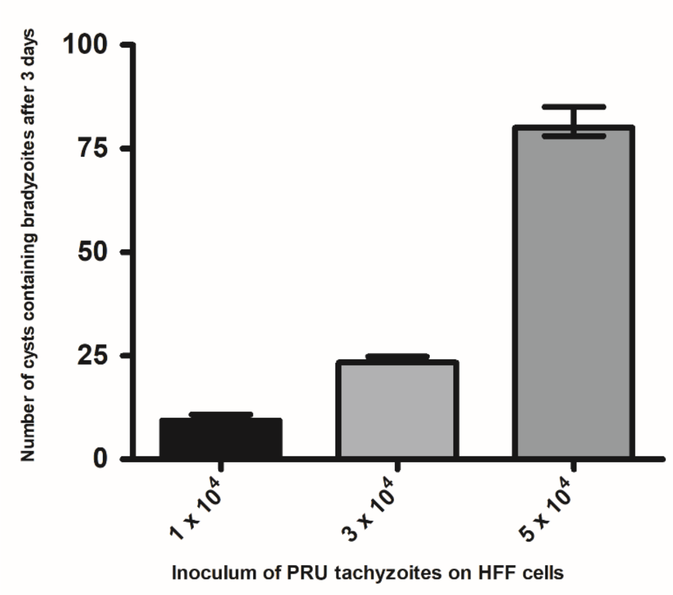

The mean number of cysts containing bradyzoites following a three-day exposure period to differentiation conditions for three inoculum concentrations: 1×10^4, 3×10^4, and 5×10^4 tachyzoites of the PRU strain of *Toxoplasma gondii*. The histogram shows the mean ± standard deviation (SD) of the values for cysts containing bradyzoites derived from independent experiments (n=3). These values were obtained by counting using zigzag scans to cover the entire glass surface from triplicate coverslips cultured of the slide under identical conditions. Quantification of cysts containing bradyzoites zigzag scans to cover the entire glass surface of the slide following a three-day incubation period demonstrated a direct proportional relationship between the number of tissue cysts containing bradyzoites and the initial inoculum. An inoculation with 1 x 10^4 tachyzoites resulted in an average of 9.3 tissue cysts per slide after three days. Increasing the inoculum to 3×10^4 tachyzoites resulted in an average of 23.3 tissue cysts. Finally, when HFF cells were infected with 5×10^4 parasites, the average number of tissue cysts increased to 81 ($r^2 = 0.88$; Pearson's test for linearity, $p = 0.0002$).

Supplementary Figure S3: FTIR compound 9

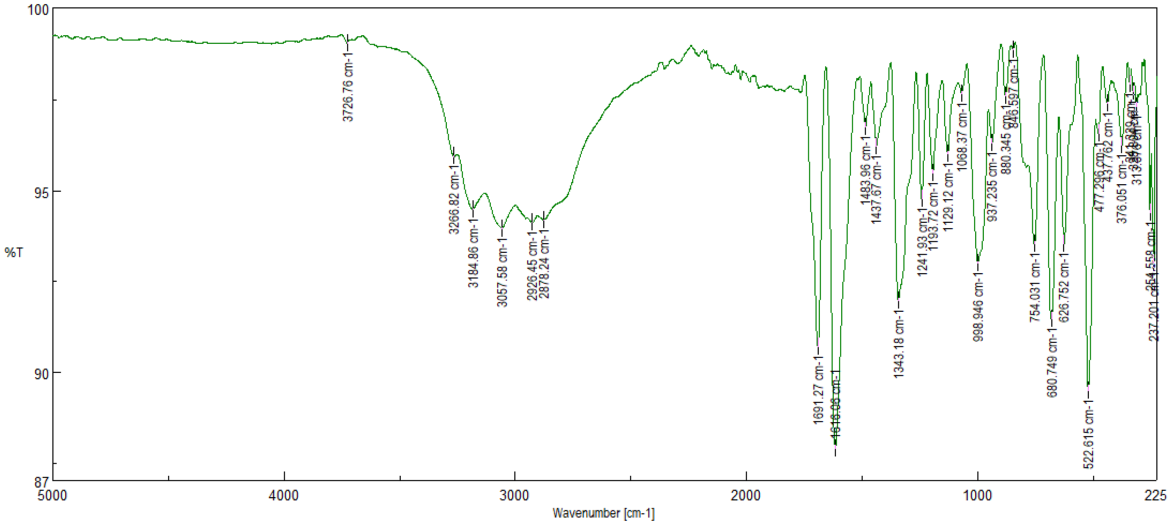

Supplementary Figure S4: FTIR compound 10

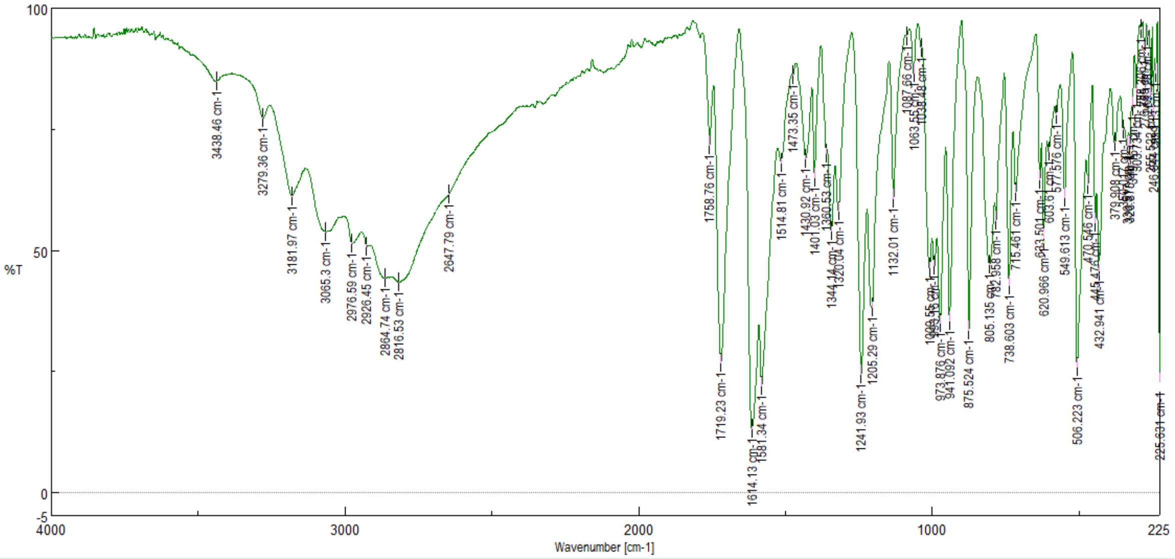

Supplementary Figure S5: FTIR compound 11

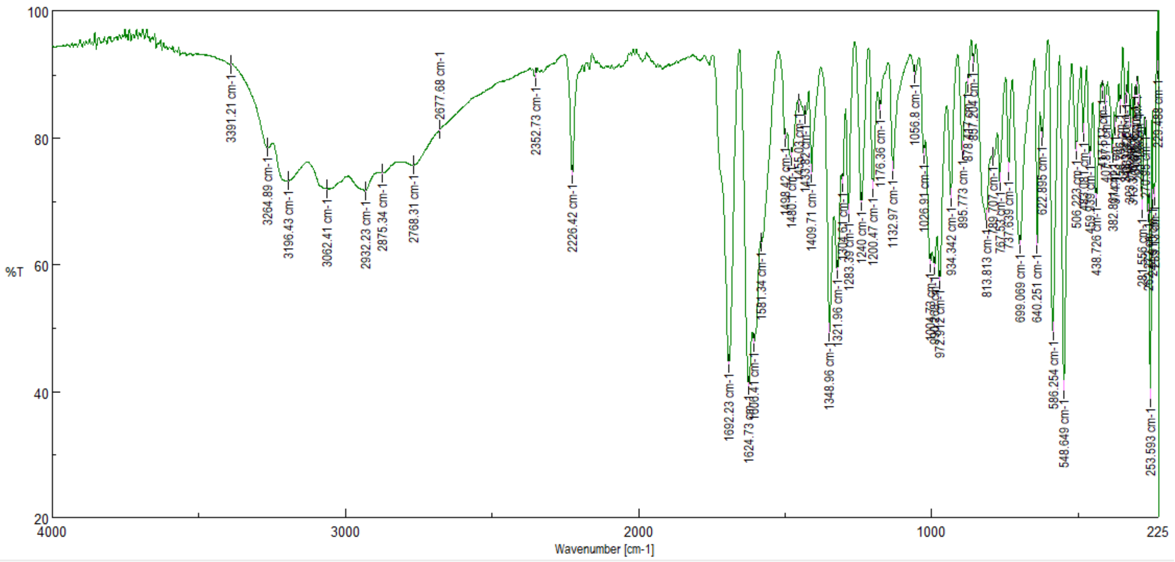

Supplementary Figure S6: FTIR compound 12

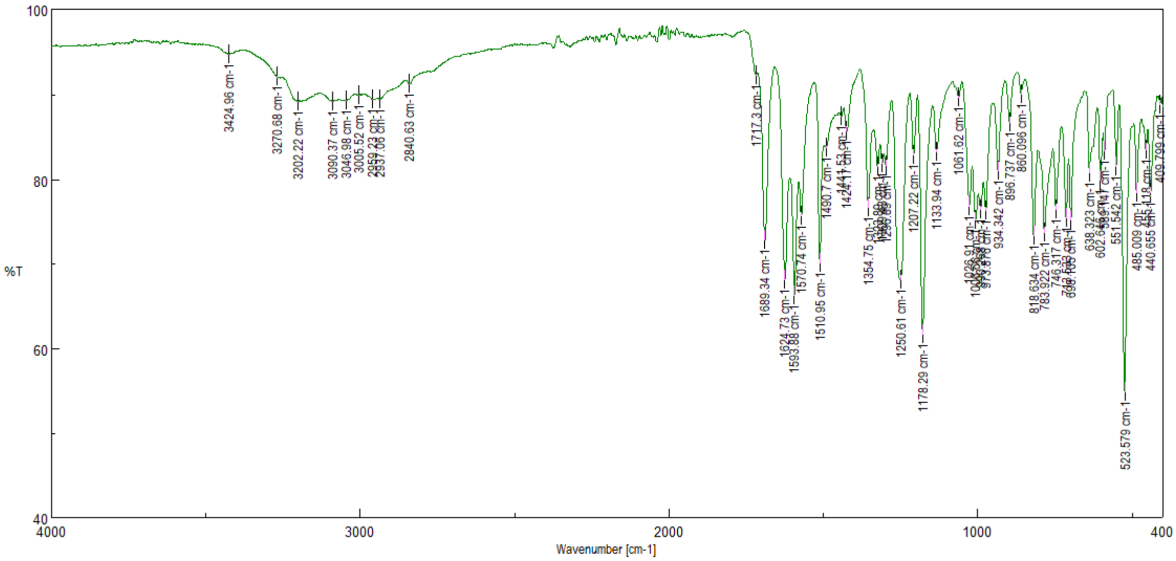

Supplementary Figure S7: FTIR compound 13

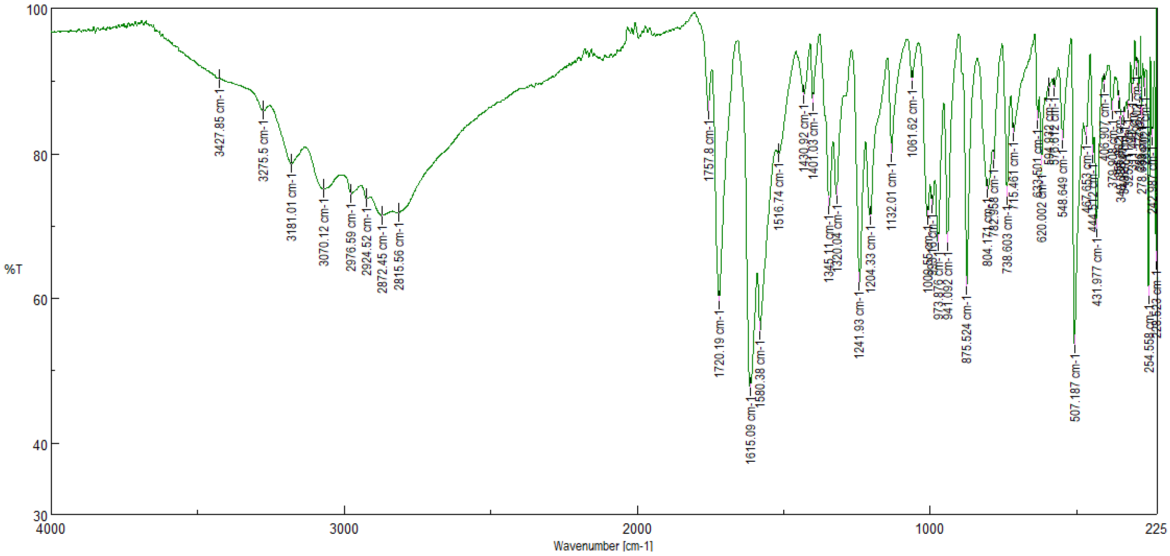

Supplementary Figure S8: FTIR compound 14

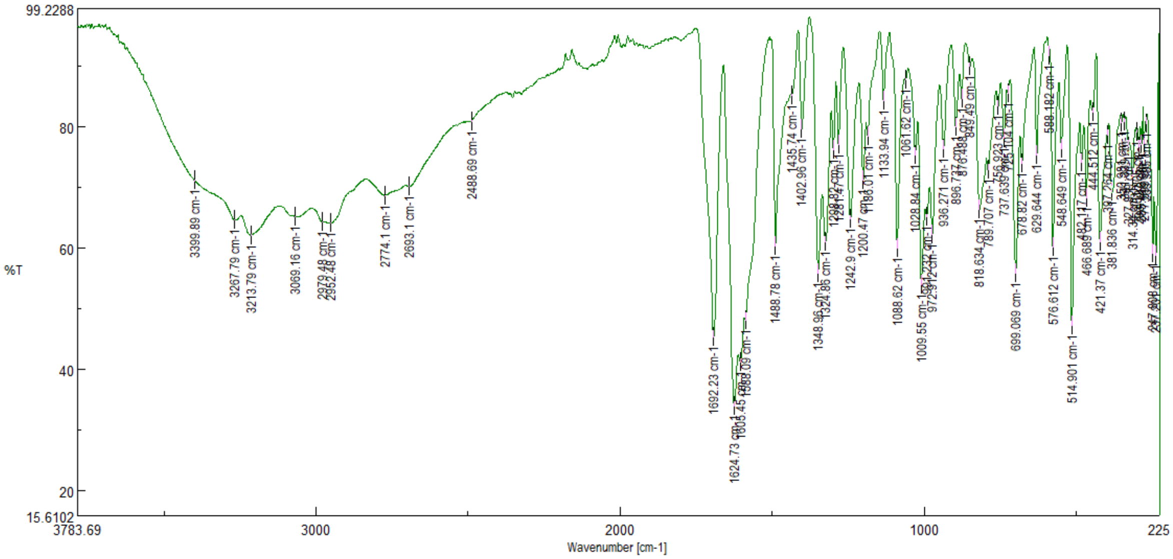

Supplementary Figure S9: ^{1}H NMR compound 15

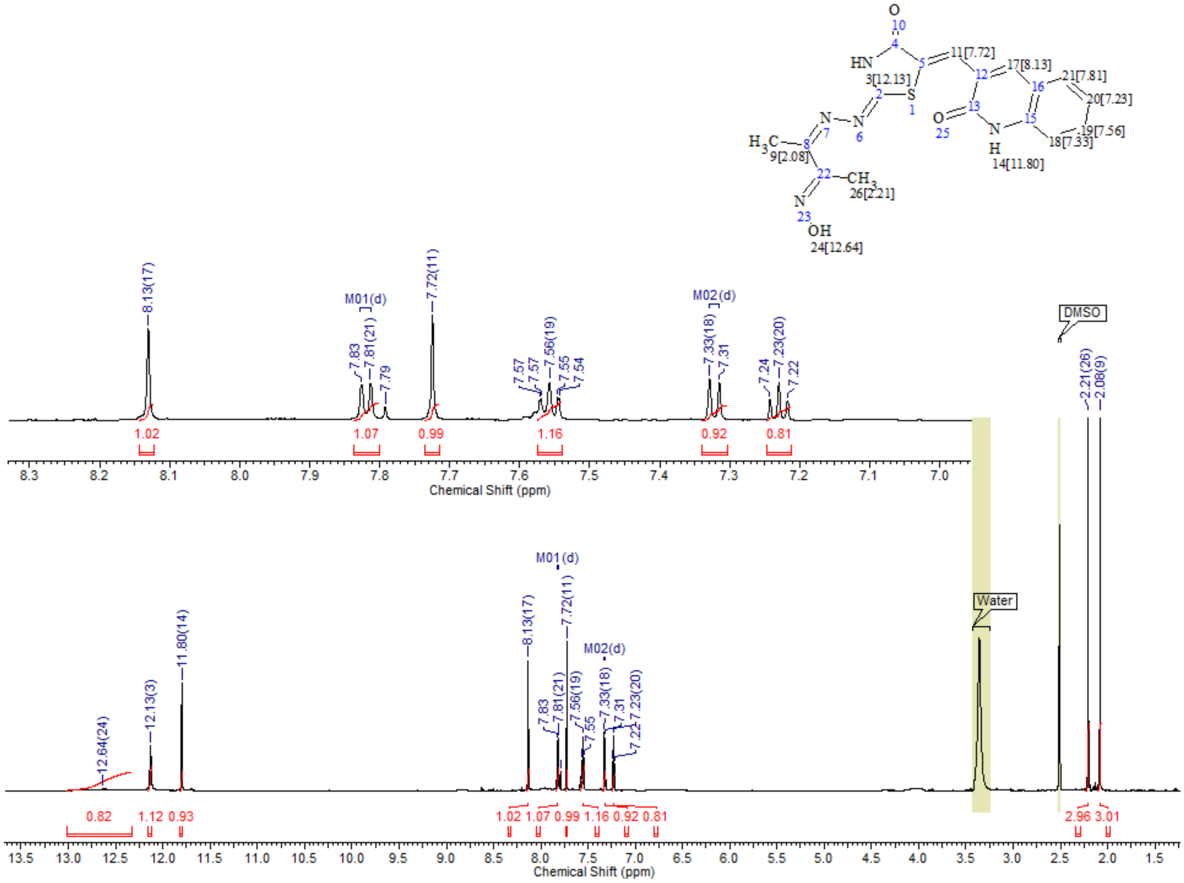

Supplementary Figure S10: ^{13}C NMR compound 15

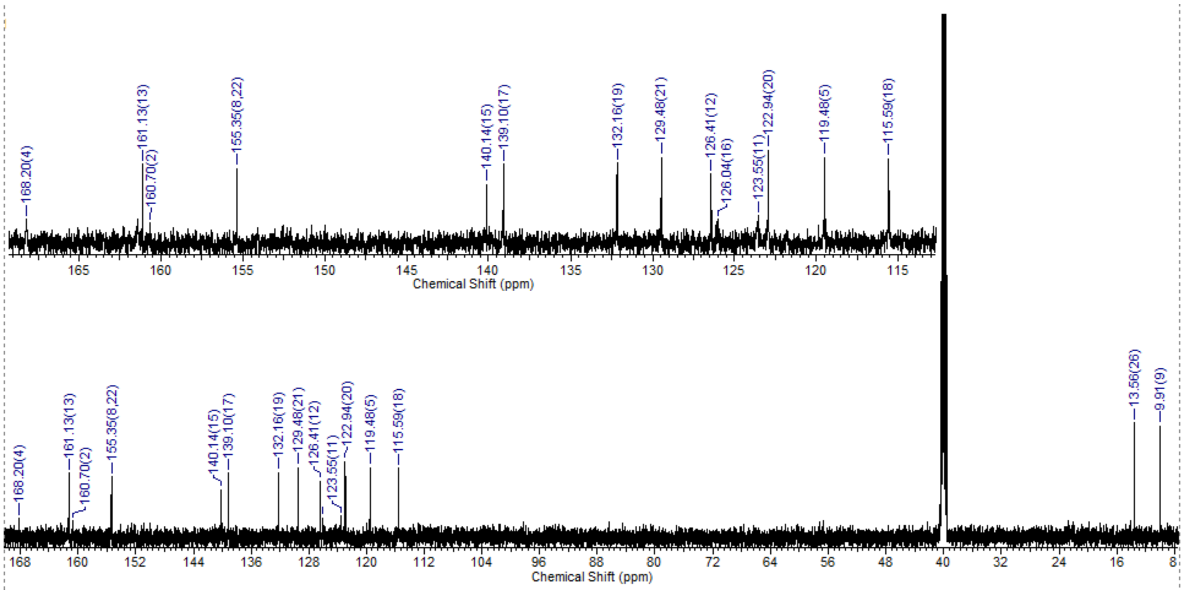

Supplementary Figure S11: FTIR compound 15

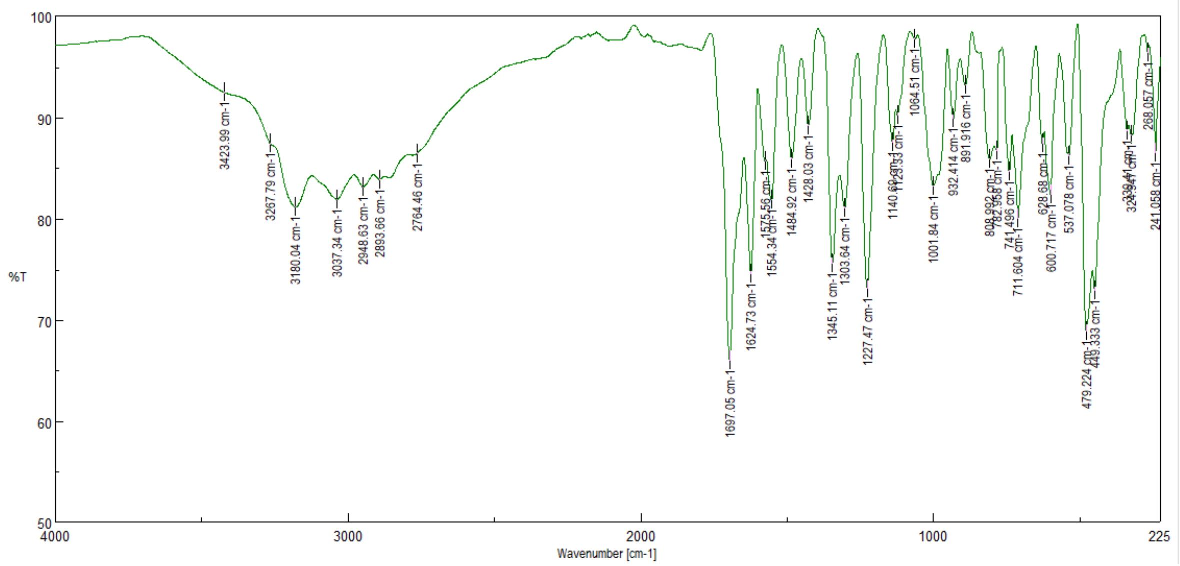

%T
Wavenumber [cm-1]
4000
3000
2000
1000
225
100
90
80
70
60
50
3423.99 cm-1
3267.79 cm-1
3180.04 cm-1
3037.34 cm-1
2948.63 cm-1
2893.66 cm-1
2764.46 cm-1
1697.05 cm-1
1624.73 cm-1
1484.92 cm-1
1428.03 cm-1
1345.11 cm-1
1303.64 cm-1
1227.47 cm-1
1064.51 cm-1
1001.84 cm-1
932.414 cm-1
891.916 cm-1
741.496 cm-1
711.604 cm-1
628.68 cm-1
600.717 cm-1
537.078 cm-1
479.224 cm-1
449.333 cm-1
268.057 cm-1
241.058 cm-1
